# ARC‐18 Improved Motor Performance Through Inhibiting ACLY‐Mediated Smad2/3 Acetylation in a Model of Duchenne Muscular Dystrophy

**DOI:** 10.1002/jcsm.70081

**Published:** 2025-10-07

**Authors:** Chongyang Chen, Bingge Zhang, Chao Yang, Jing Wang, Ye He, Haitao Yu, Jianjun Liu, Yongmei Xie, Xifei Yang, Gong‐Ping Liu

**Affiliations:** ^1^ Department of Pathophysiology, School of Basic Medicine, Key Laboratory of Ministry of Education of China and Hubei Province for Neurological Disorders Tongji Medical College, Huazhong University of Science and Technology Wuhan China; ^2^ National Health Commission Key Laboratory of Nuclear Medicine, Jiangsu Key Laboratory of Molecular Nuclear Medicine Jiangsu Institute of Nuclear Medicine Wuxi China; ^3^ Shenzhen Key Laboratory of Modern Toxicology, Shenzhen Medical Key Discipline of Health Toxicology (2020–2024) Shenzhen Center for Disease Control and Prevention Shenzhen China; ^4^ State Key Laboratory of Biotherapy and Cancer Center, West China Hospital Sichuan University and Collaborative Innovation Center of Biotherapy Chengdu China; ^5^ Co‐Innovation Center of Neuroregeneration Nantong University Nantong Jiangsu China

**Keywords:** ACLY, ARC‐18, Duchenne muscular dystrophy, motor performance, proteomics

## Abstract

**Background:**

Duchenne muscular dystrophy (DMD) is a genetic disorder characterized by progressive muscle weakness, with inflammation and fibrosis contributing to its pathogenesis. Despite advancements in genetic disease‐modifying treatment, there is currently no effective pharmacological treatment for DMD.

**Methods:**

New compound ARC‐18, a derivative of Arctigenin known for its anti‐inflammatory activity, was designed and synthesized in our lab and administered prophylactically to 2‐month‐old mdx mice for 60 days. The motor performance was investigated by rotarod test, climbing‐pole test, grip strength test, hanging endurance test, treadmill endurance test and gait analysis. Afterwards, molecular biological experiments, including proteomics, immunohistochemistry, immunofluorescence, western blots, gene transfection and immunoprecipitation, were employed to investigate the molecular mechanism of ARC‐18 in the treatment of mdx.

**Results:**

ARC‐18 significantly ameliorated the motor performance of DMD mice (rotating time +65.9%, *p* < 0.01; hanging time +59.7%, *p* < 0.05; grip strength +32.1%, *p* < 0.0001; climbing time −29.0%, *p* < 0.0001; numbers of electric shocks −69.3%, *p* < 0.01) by up‐regulating the expression of dystrophin‐associated proteins (dystrophin, *p* < 0.01; α‐dystroglycan, *p* < 0.01) and down‐regulating the expression of muscle satellite/stem cell proteins (Pax7, *p* < 0.05; Myod, *p* < 0.05; Myog, *p* < 0.05; α‐SMA, *p* < 0.01; fibronectin, *p* < 0.001; collagen I, *p* < 0.05). ARC‐18 prevented the progression of muscle fibrosis, reduced inflammatory factors transforming growth factor (TGF) β1 (*p* < 0.05), IL‐1β (*p* < 0.05) and TNF‐α (*p* < 0.05) levels, and promoted the structural integrity of gastrocnemius and triceps muscles. Proteomics analysis demonstrated that ARC‐18 treatment reversed the protein expression pattern of DMD model mice, with ATP‐citrate synthase (ACLY) enriched in the TCA cycle pathway, showing a significant correlation with DMD expression levels (*R* = −0.72, *p* = 0.00031). Further investigations revealed that ARC‐18 directly bound with ACLY (EC_50_ = 120.2 nM) to promote its degradation by the proteasome system and suppressed the ACLY‐mediated acetylation of Smad2/3 (*p* < 0.01) to reduce its nuclear localization (*p* < 0.05) to inhibit fibrosis.

**Conclusions:**

Our study indicated that oral ARC‐18 treatment decelerated the progression of neuromuscular disease in a reliable DMD animal model, suggesting its potential as a promising drug for DMD.

**Abbreviations**
ACLY: ATP‐citrate lyase
CK: Creatine kinase
DFZ: Deflazacort
DMD: Duchenne muscular dystrophy
eMyHC: Embryonic myosin heavy chain
MHC: Myosin heavy chain, muscle
Myod: Myoblast determination protein
Myog: Myogenin
Pax7: Paired box protein Pax‐7
Smad2/3: Mothers against decapentaplegic homolog 2/3
TGF β1: Transforming growth factor (TGF) β1
α‐SMA: α‐smooth muscle actin

## 1 Introduction

Duchenne muscular dystrophy (DMD) is a severe, progressive, atrophic X‐linked genetic disorder that causes difficulties with movement and ultimately leads to breathing difficulties and premature death [1]. This disease is induced by a lack of functional dystrophin protein, which leads to progressive muscle degeneration. Previous studies have shown that the gradual loss of muscle fibres, accompanied by increased inflammation and fibrosis, contributes to progressive muscle deterioration in DMD [2, 3]. Although the gene therapy that can restore the expression of partially functional dystrophin protein is a promising approach, the expression of the full‐length dystrophin gene, which exceeds the packaging limits of the viral vector, is the most challenging aspect of gene therapy [4]. Therefore, it is necessary to find new molecular drugs that can remedy DMD effectively.

Skeletal muscle fibrosis is a characteristic injury associated with DMD and is defined as muscle degeneration, which is followed by loss of mobility [5]. Fibroblasts are a pivotal mediator of fibrosis and remain persistently activated, leading to extensive collagen deposition in DMD [6]. During chronic injury, fibroblasts undergo activation in response to transforming growth factor (TGF) β1 [7]. Numerous studies have demonstrated a significant elevation of TGF β1 levels in the muscles of individuals with DMD, prompting the exploration of TGF β1 inhibition or inactivation of its signalling pathway as a potential strategy to mitigate fibrosis in DMD [8, 9]. Nevertheless, currently there are no clinically effective drugs specifically targeting TGF β1 or its downstream effectors available for attenuating fibrosis in DMD.

Arctigenin, which has been reported to have pharmacological activity of anti‐inflammation, antioxidative stress and antifibrosis, etc. [10], can repress TGF β stimulated epithelial mesenchymal transition signals in human lung cancer cells [11] and decrease the expression of TGF β1 in bleomycin‐induced fibrotic skin, followed by suppressing fibroblast activity and extracellular matrix deposition [12]. Moreover, Arctigenin has been confirmed to reduce myofibroblast activities in oral submucous fibrosis via the reduction of TGF β expression [13]. However, Arctigenin exhibits limited clinical potential due to its extremely poor solubility in water, which severely restricts bioavailability [14]. Consistent with prior studies showing that amino acid ester or glucuronide derivatives substantially improve water solubility and in vivo activity over Arctigenin [15], we designed and synthesized a novel small‐molecule prodrug, ARC‑18, to enhance pharmacokinetic properties. ARC‑18 was rationally engineered by our team to improve aqueous solubility and in vivo delivery compared to parent Arctigenin.

Here, we investigated the therapeutic effects of ARC‐18 in mdx mice, a gene editing model of human DMD. We discovered that chronic oral therapy with ARC‐18 improved locomotion function of mdx mice by direct binding with ACLY to promote its degradation, and reduced the ability of ACLY acetylation of Smad2/3 to impede Smad2/3 transport into the nucleus to suppress skeletal muscle fibrosis.

## 2 Methods

### 2.1 Regents and Antibodies

ARC‐18 was designed and synthesized from our laboratory to reach a stated purity ≥ 99.2%. The structure and information were provided in Supplementary Figure 1A. In this study, the used antibodies and chemical reagents have been presented in Supplementary Table 1.

### 2.2 Animal Treatment

The male DMD model mice (C57BL/10ScSnJNju‐Dmd^em3Cd4^/Gpt (mdx)) and corresponding control mice (C57BL/10ScSn/J (WT)) were purchased from GemPharmatech (Nanjing, China). All the animals were placed in a 12‐h light and dark cycle room with stable temperature (20°C ± 2°C) and humidity (55 ± 5%). To assess the compound’s safety profile and potential physiological effects in non‐dystrophic animals. The WT mice aged 8 weeks were oral gavage with 32.9 mg/kg (high dose, abbreviated as High) ARC‐18 or the same volume of corresponding vehicle daily for 2 months. The mdx mice aged 8 weeks were oral gavage with 11 mg/kg (low dose, abbreviated as low) or 32.9 mg/kg (high dose) ARC‐18, or 1 mg/kg deflazacort (DFZ, intraperitoneal injection), or the same volume of corresponding vehicle daily for 2 months. All animal experiments were performed as following the ‘Animals and Human Use Policy’ and the Guidelines for the Care and Use of Laboratory Animals issued by the Ministry of Science and Technology. The Animal Care and Use Committee of the Shenzhen Center for Disease Control and Prevention approved the study protocol. The behavioural and pathological methods were described in detail in the supplementary materials.

### 2.3 Evans Blue Staining

The integrity of gastrocnemius and triceps was evaluated by Evans blue dye (EBD) and suffered from the previous research [16]. Briefly, 24 h before euthanasia, 1% EBD with a dose of 1% volume to body weight was injected into the peritoneal cavity. Upon euthanasia, the experimenter removed the hind leg skin and photographed skeletal muscles; if shown as blue coloration, it indicated dye uptake into the muscles.

### 2.4 Blood Biochemical Analysis

After the behavioural test, the blood samples were collected from the inferior vena cava, placed at room temperature for 1 h, and then centrifuged for 10 min at 1500 g to obtain serum. The CK activity and CLU, alanine aminotransferase (ALT), creatinine (Cr), aspartate aminotransferase (AST) and blood urea nitrogen (BUN) levels in serum were assayed by HITACHI7080 Automatic Clinical Analyser (Tokyo, Japan).

### 2.5 Inflammation Assay

According to the manufacturer's instructions, an enzyme‐linked immunosorbent assay (ELISA) was used to detect the levels of IL‐1β, TNF‐α and TGF‐β in gastrocnemius. The mouse ELISA kit came from Elabscience‐Wuhan Elite Biological Co. Ltd.

### 2.6 Cell Culture and Gene Transfection

The C2C12 cell was cultured in a 37°C temperature incubator with 5% CO_2_. For ACLY gene transfection, the protocol was referenced in a previous study [17]. The information on the plasmid pcDNA3.1(+)‐3xFLAG is as follows: Briefly, the C2C12 cell was inoculated in 6‐well plates until about 80% full and then transfected with the overexpressed plasmid of ACLY for 48 h via lipofectamine 2000 reagent. After that, the C2C12 cell was collected for further study.

### 2.7 Immunohistochemistry Staining

Mice were anaesthetized with 2% sodium pentobarbital and subjected to cardiac perfusion with normal saline. Bilateral gastrocnemius muscles were surgically excised and immediately fixed in 4% paraformaldehyde solution for 24 h. Paraffin‐embedded sections (5 μm) were prepared using a microtome (Leica, Germany) for subsequent histological analyses. Briefly, deparaffinized and rehydrated sections underwent antigen retrieval in citrate buffer (pH 6.0). Endogenous peroxidase activity was quenched with 3% H₂O₂ treatment for 10 min. Sections were blocked with 3% bovine serum albumin (BSA) for 1 h, followed by overnight incubation with primary antibodies at 4°C. Biotinylated goat antipolyvalent secondary antibodies (DAB Kit, ab64264, Abcam, USA) were applied for 1 h, followed by streptavidin–peroxidase conjugate incubation for 1 h. Images were acquired using the Leica Aperio GT 450 Digital Pathology System (Leica, Germany).

### 2.8 Molecular Docking

The crystal structure of ACLY (PDB ID: 6HXK) was downloaded from the Protein Data Bank (https://www.rcsb.org/). The 2D and 3D structure of ARC‐18 was generated and optimized by Molecular Operating Environment (MOE, 2022.02). Molecular docking was performed three times using AutoDock Vina (v1.2.3), and the top binding degree was recorded. After docking, the best binding pose was visualized and analyzed by PyMOL (v2.6.0) and MOE (2022.02).

### 2.9 Cellular Thermal Shift Assay

C2C12 cells were seeded in six‐well plates, adhered, and incubated with DMSO or different concentrations of ARC‐18 (10 nm, 50 nm, 100 nm, 500 nm, 1 μm, 5 μm, 10 μm, 50 μm, 100 μm) for 24 h, and co‐cultured with 5 ng/mL of TGF‐β for 12 h. Cells were digested with trypsin, rinsed with PBS, and the supernatants were centrifuged and discarded. The supernatant was centrifuged and discarded. The supernatant of each group was resuspended in PBS with protease inhibitors, placed at 55°C for 3 min, and then equilibrated at RT for 3 min. Cells were lysed by three cycles of liquid nitrogen snap‐freezing and thawing. The lysate was centrifuged at 20,000 × g for 20 min, and the supernatant was mixed with 5× loading buffer for subsequent Western blot analysis.

### 2.10 Statistical Analysis

The data were analyzed using GraphPad Prism 9.0 statistical software (GraphPad Software, Inc., La Jolla, CA, USA). In the Figures, behavioral data were presented as mean ± SEM, whereas all other types of data were depicted as mean ± SD. For comparison between the two groups, the Student *t* test and one‐way ANOVA or two‐way repeated measures ANOVA followed by the Dunnett test, was used to analyze multiple comparisons. *p* < 0.05 was set as the significant difference between groups. The correlation analysis was performed using the ‘Hmisc’ package within the RStudio environment.

## 3 Results

### 3.1 Administration of ARC‐18 Improved the Motor Performance of mdx Mice.

**FIGURE 1:**
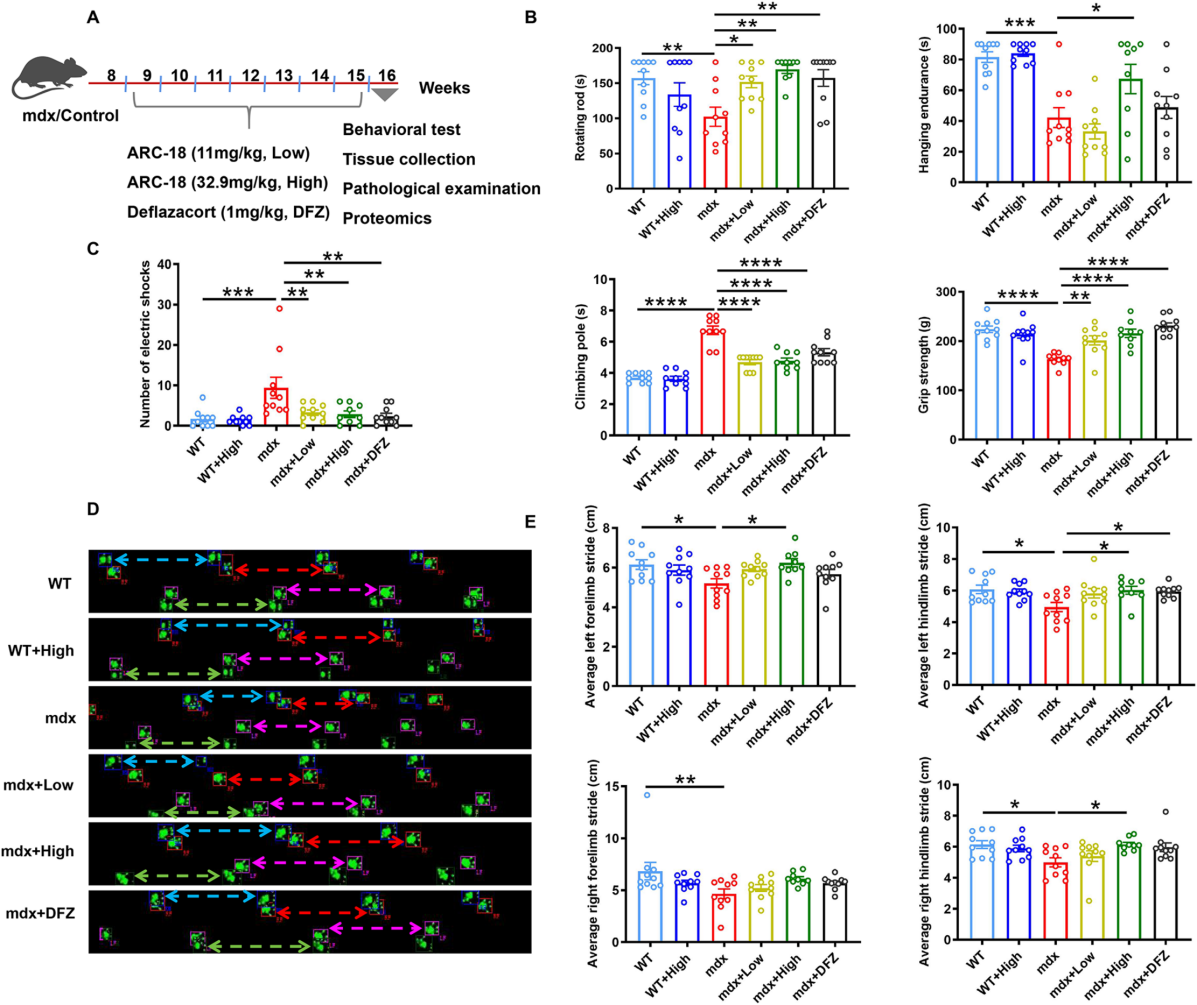
ARC‐18 administration improved motor performances of mdx mice. Two‐month‐old mdx mice were administrated with ARC‐18 for 8 weeks, and then motor performance was detected. (A) The schedule of experiment mice treated with drugs and experimental time point. (B)The motor performance of retention time on rotarod via rotarod test, hanging endurance time via hanging endurance test, grip strength measured via grip strength test, climbing time on pole tests via climbing‐pole test, (C) number of electric shocks via treadmill endurance test, (D) the representative images of gait tracking via gait analysis and (E) quantified average stride of lift hindlimbs and forelimbs or right hindlimbs and forelimbs. Data were shown as mean ± SEM. *, *p* < 0.05, **, *p* < 0.01, ***, *p* < 0.001, ****, *p* < 0.0001. *n* = 10 for each group.

The phenotypes of the DMD model (mdx mice), such as muscle fibre atrophy and inflammatory cell infiltration, were observed at 8 weeks [18]. The average plasma concentrations of ARC‐18 and Arctigenin with an equimolar ratio at 1 h after oral administration were 24.66 and 8.52 ng/mL, respectively (Data were not shown.). To evaluate the therapeutic effects of ARC‐18, we orally administered ARC‐18 to 2‐month‐old mdx mice for 8 weeks with a dose of 11 (low dose, abbreviated as low), or 32.9 mg/kg (HIGH DOSE, ABBREVIATED AS HIGH, the high dose was set based on one‐tenth of the LD_50_), with control mice (mdx and WT) receiving an equivalent volume of vehicle for the same term. Meanwhile, the DFZ, daily intraperitoneal injection with 1 mg/kg, was set as a positive therapeutic drug (Figure 1A). During the treatment, the weight of all the experimental mice gradually increased, while the weight between each therapeutic group and their control showed no difference; the only significant change observed was in the compared group of mdx versus WT (32.8 ± 0.64 g mdx mice compared with 28.5 ± 0.57 g WT mice, *p* < 0.0001) (Supplementary Figure 1B). After excluding that the drug treatment had no effect on the body weight of the mice, the motor behavioural performances of the rotating rod, hanging endurance, grip strength, climbing pole and treadmill endurance (indicated by numbers of electric shocks) were assessed. Consistent with a previous study [19], the mdx mice showed significant deterioration of motor ability in all the motor behavioural tests compared with WT mice (rotating time −34.9%, *p* < 0.01; hanging time −48.3%, *p* < 0.001; grip strength −27.2%, *p* < 0.0001; climbing time +82.0%, *p* < 0.0001; numbers of electric shocks +452.9%, *p* < 0.001). ARC‐18 treatment significantly improved motor performance in mdx mice, with the high dose ameliorating most motor defects (rotating time +65.9%, *p* < 0.01; hanging time +59.7%, *p* < 0.05; grip strength +32.1%, *p* < 0.0001; climbing time −29.0%, *p* < 0.0001; numbers of electric shocks −69.3%, *p* < 0.01) (Figure 1B,C). Both ARC‐18 and DFZ treatments enhanced functional outcomes, and while high‐dose ARC‐18 treated mice showed a trend toward better performance in hanging endurance compared to DFZ treated mice (hanging time +7.9%), this difference did not reach statistical significance (Figure 1B). The gait analysis can be used for evaluating the motor coordination ability of mdx mice after ARC‐18 treatment. In this experiment, the average stride of climbs was recorded to assess motor coordination. Compared with WT mice, the average left forelimb and hindlimb stride, or average right forelimb and hindlimb stride of mdx mice were significantly decreased (left forelimb stride −0.95 ± 0.31 cm, *p* < 0.05; left hindlimb stride −1.11 ± 0.34 cm, *p* < 0.05; right forelimb stride −2.19 ± 0.63 cm, *p* < 0.01; right hindlimb stride −1.15 ± 0.37 cm, *p* < 0.05) The high dose of ARC‐18 administration significantly increased the average stride except for the right forelimb (compared with mdx mice, left forelimb stride +1.04 ± 0.32 cm, *p* < 0.05; left hindlimb stride +1.07 ± 0.35 cm, *p* < 0.05; right forelimb stride +1.47 ± 0.65 cm, p = ns; right hindlimb stride +1.16 ± 0.38 cm, *p* < 0.05), while DFZ could only significantly increase the average left hindlimb stride (+1.00 ± 0.34 cm, *p* < 0.05) (Figure 1D, E). In addition, the high dose of ARC‐18 treatment in WT mice showed no difference in all motor behavioural tests compared with untreated WT mice. These data indicated that ARC‐18 ameliorated the motor performance of mdx mice.

### 3.2 ARC‐18 Treatment Promoted the Muscular Integrity and Suppressed Pathological Changes of mdx Mice

**FIGURE 2:**
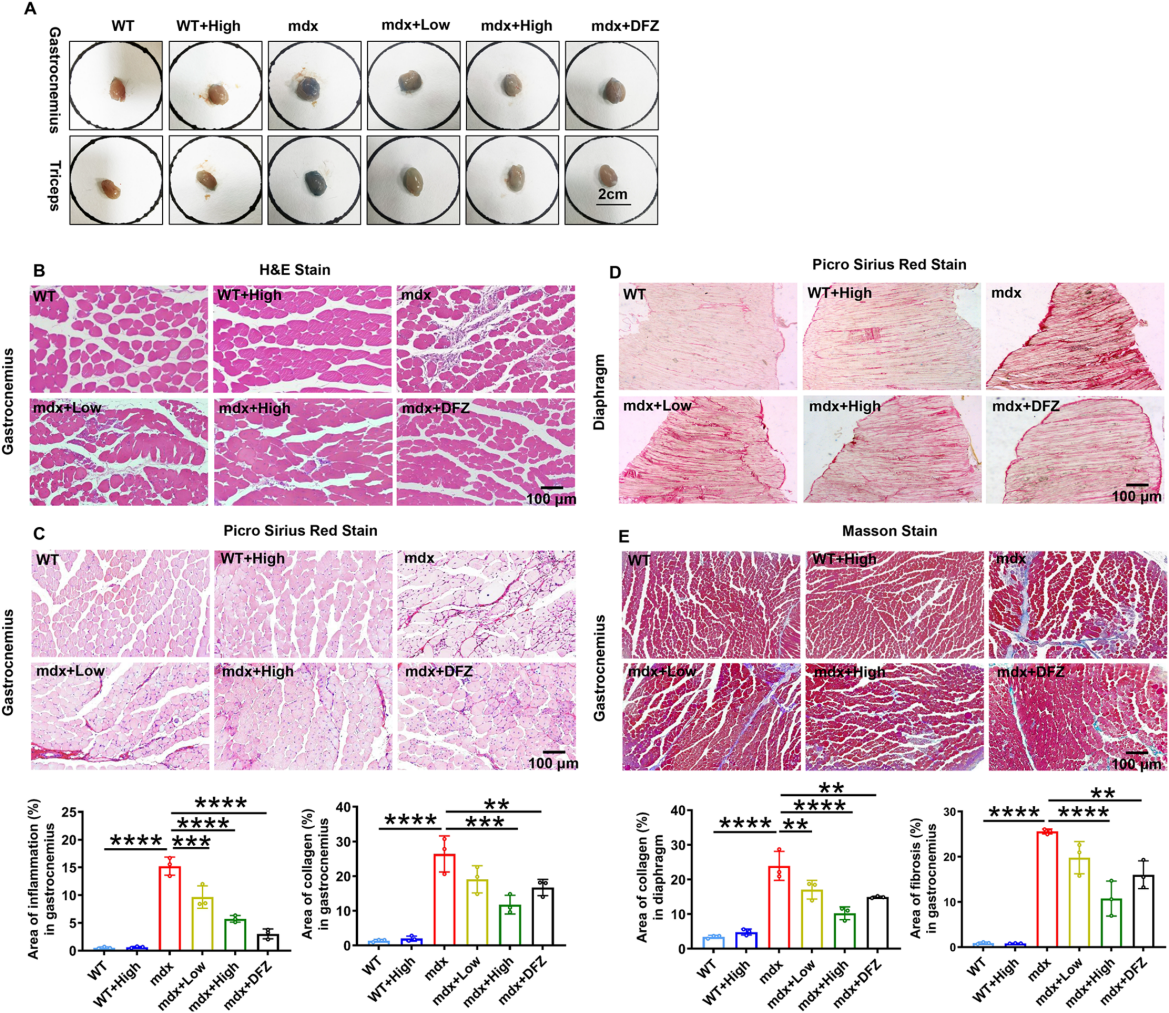
ARC‐18 treatment reduced muscle damage and suppressed pathologic deterioration of mdx mice. (A) Representative images of gastrocnemius and triceps in experimental mice after intraperitoneal injection of Evans blue stride. (B) H&E stain of gastrocnemius in all the experimental mice and quantification of inflammation area. The Picro Sirius Red Stain and quantification of collagen area in gastrocnemius (C) and diaphragm (D). (E) Masson Stain and quantification for detection of collagen fibres in gastrocnemius after ARC‐18 treatment in mdx mice. Data were shown as mean ± SD. *, *p* < 0.05, **, *p* < 0.01, ****, *p* < 0.0001. *n* = 3 for each group.

After behavioural tests, a part of experiment mice was intraperitoneally injected with Evans blue dye (EBD) to assess the muscular integrity. As shown in Figure 2A, the permeability of Evans blue in the gastrocnemius and triceps muscles was obviously reduced after ARC‐18 or DFZ treatment. Possibly due to the short treatment time or the mice being too young, the hypertrophy of the gastrocnemius (0.36 ± 0.015 g high‐dose ARC‐18–treated mdx mice, 0.37 ± 0.007 g DFZ‐treated mdx mice) and triceps (0.35 ± 0.013 g high‐dose ARC‐18–treated mdx mice, 0.33 ± 0.006 g DFZ‐treated mdx mice) was not alleviated after ARC‐18 or DFZ treatment, and cardiac hypertrophy was not observed in mdx mice compared with WT mice (0.13 ± 0.006 g and 0.15 ± 0.006, respectively) (Supplementary Figure 2A). In DMD patients, fibrosis is a major reason for muscle weakness [20], high dose of ARC‐18 significantly reduced the inflammation area (from 15.2 ± 1.6% to 5.7 ± 0.60%, *p* < 0.0001) and collagen fiber hyperplasia (from 26.4 ± 5.2% to 11.8 ± 2.7%, *p* < 0.001) of gastrocnemius in mdx mice (Figure 2B,C), and obviously suppressed the fibrosis and degeneration of the diaphragm (from 23.9 ± 4.2% to 10.2 ± 1.9%, *p* < 0.0001) (Figure 2D). Another staining method for collagen fibers (Masson stain) also showed a diminished fibrosis of gastrocnemius and heart in ARC‐18 treated mdx mice (Figure 2E, Supplementary Figure 2B).

In addition, ARC‐18 significantly reduced the creatine kinase (CK) activity in serum of mdx mice (from 9121 ± 1051 U/L to 5626 ± 2140 U/L, *p* < 0.01), which implies the protective effect against muscle damage. We also assayed the levels of glucose (GLU), ALT, aspartate aminotransferase (AST), Cr and BUN in serum to assess the health status after ARC‐18 treatment and found that ARC‐18 significantly reduced the serum Cr of mdx mice (Supplementary Figure 3A). Inflammation is a pathological character of DMD and contributes to the deterioration of DMD. ARC‐18 (high dose) treatment significantly decreased the inflammatory factors IL‐1β (from 14.6 ± 1.1 pg/mg protein to 11.5 ± 2.0 pg/mg protein, *p* < 0.05), TNF‐α (from 19.8 ± 1.8 pg/mg protein to 16.2 ± 2.0 pg/mg protein, *p* < 0.05) and TGF β1 (from 0.37 ± 0.10 ng/mg protein to 0.25 ± 0.07 ng/mg protein, *p* < 0.05) in mdx mice and showed a better reversible effect than DFZ treatment (Supplementary Figure 3B). All these data reflected that ARC‐18 protected against the muscular damage in mdx mice.

### 3.3 ARC‐18 Improved Myoblasts' Quality and Inhibited the Phenotypic Conversion of Myoblasts Into Fibroblasts in mdx Mice

**FIGURE 3:**
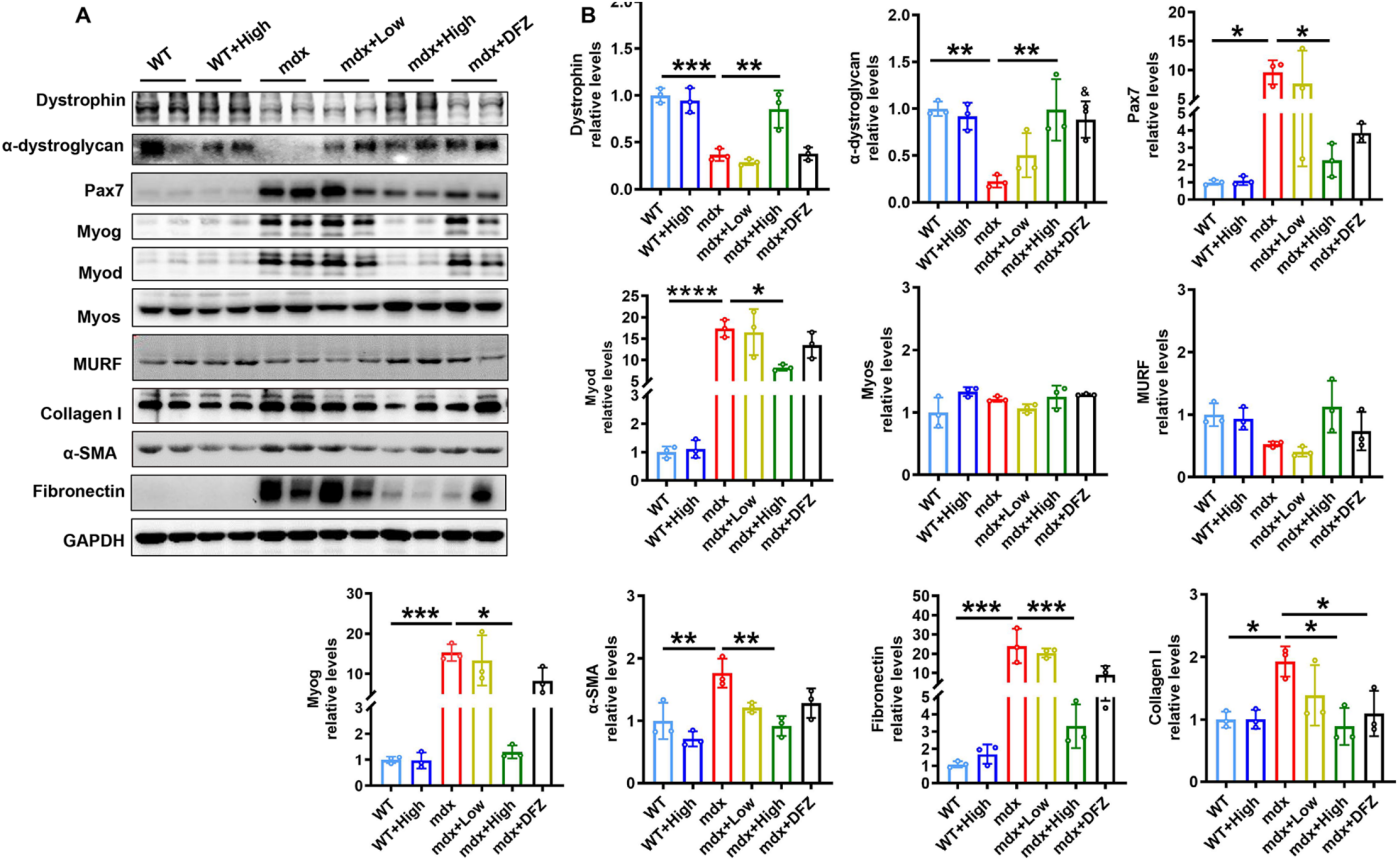
ARC‐18 administration suppressed abnormal myoblast phenotypic conversion in mdx mice. (A) Western blot of proteins associated with muscle function and fibrosis. (B) Quantification of the muscle atrophy marker Dystrophin, the structural protein α‐dystroglycan, the stemness marker Pax7, the early myogenic marker Myod, the myofunctional protein Myos, the muscle atrophy reflected protein MURF, the differentiation marker Myog and the collagen fibrinogenic protein of α‐SMA, fibronectin and collagen I in gastrocnemius of all experimental mice. Data were shown as mean ± SD. *, *p* < 0.05, **, *p* < 0.01, ***, *p* < 0.001, ****, *p* < 0.0001. *n* = 3 for each group.

Following ARC‐18 treatment, we assessed the expression of molecules associated with DMD clinical features in the gastrocnemius muscle. High doses of ARC‐18 significantly increased the expression of the structural protein dystrophin and α‐dystroglycan, indicating that ARC‐18 promoted the maintenance of proper muscle morphology (Figure 3B). The inhibition of differentiation is partly induced by DMD atrophy; we examined myoblast differentiation and fibrosis in ARC‐18 treated mdx mice. Western blot analysis revealed an increased expression of the stemness marker Pax7, early myogenic marker Myod and differentiation marker Myog in mdx mice. However, the myofibroblast markers alpha‐smooth muscle actin (α‐SMA) and collagen I were also significantly increased in mdx mice (Figure 3). These results indicated that mdx mice showed a phenotypic conversion of myoblasts into fibroblasts, while the administration of ARC‐18 can significantly reverse this phenotypic conversion and suppress the production of fibrosis, reflected by decreased expression of Pax7, Myod, Myog, α‐SMA, collagen I and fibronectin. Another myoblast differentiation protein, myostatin (Myos), muscle atrophy protein and muscle RING‐finger (MURF) were not changed in ARC‐18 treated or untreated mice compared with their control (Figure 3B).

In addition, immunofluorescence and immunohistochemistry showed that ARC‐18 obviously increased the structural protein of Laminin (Supplementary Figure 4A, B), the myogenic marker protein of myosin heavy chain (MHC) (Supplementary Figure 4E, F) in gastrocnemius (+40.7%, *p* < 0.01) and diaphragm (+87.6%, p = ns) of mdx mice. The embryonic myosin heavy chain (eMyHC) was also increased (+99.7%, *p* < 0.0001) in gastrocnemius of mdx mice (Supplementary Figure 4C). The immunohistochemistry of Fibronectin reconfirmed that ARC‐18 suppressed the fibrosis (−61.8%, *p* < 0.0001) in mdx mice (Supplementary Figure 4D). The increased expression of eMyHC and total MHC suggests enhanced fusion of satellite cells into regenerating myofibers. Concurrently, a significant reduction in fibrosis‐associated proteins (collagen I, α‐SMA, and fibronectin) was observed, which typically accumulate during myoblast‐to‐fibroblast phenotypic conversion. Collectively, these findings indicate that ARC‐18 promotes satellite cell activation and myogenic differentiation while inhibiting fibrotic conversion. All the results proved that ARC‐18 maintained the myoblasts quality, inhibited the phenotypic conversion of myoblasts into fibroblasts in mdx mice.

### 3.4 ARC‐18 Modified the Protein Expression Profile in mdx Mice

**FIGURE 4:**
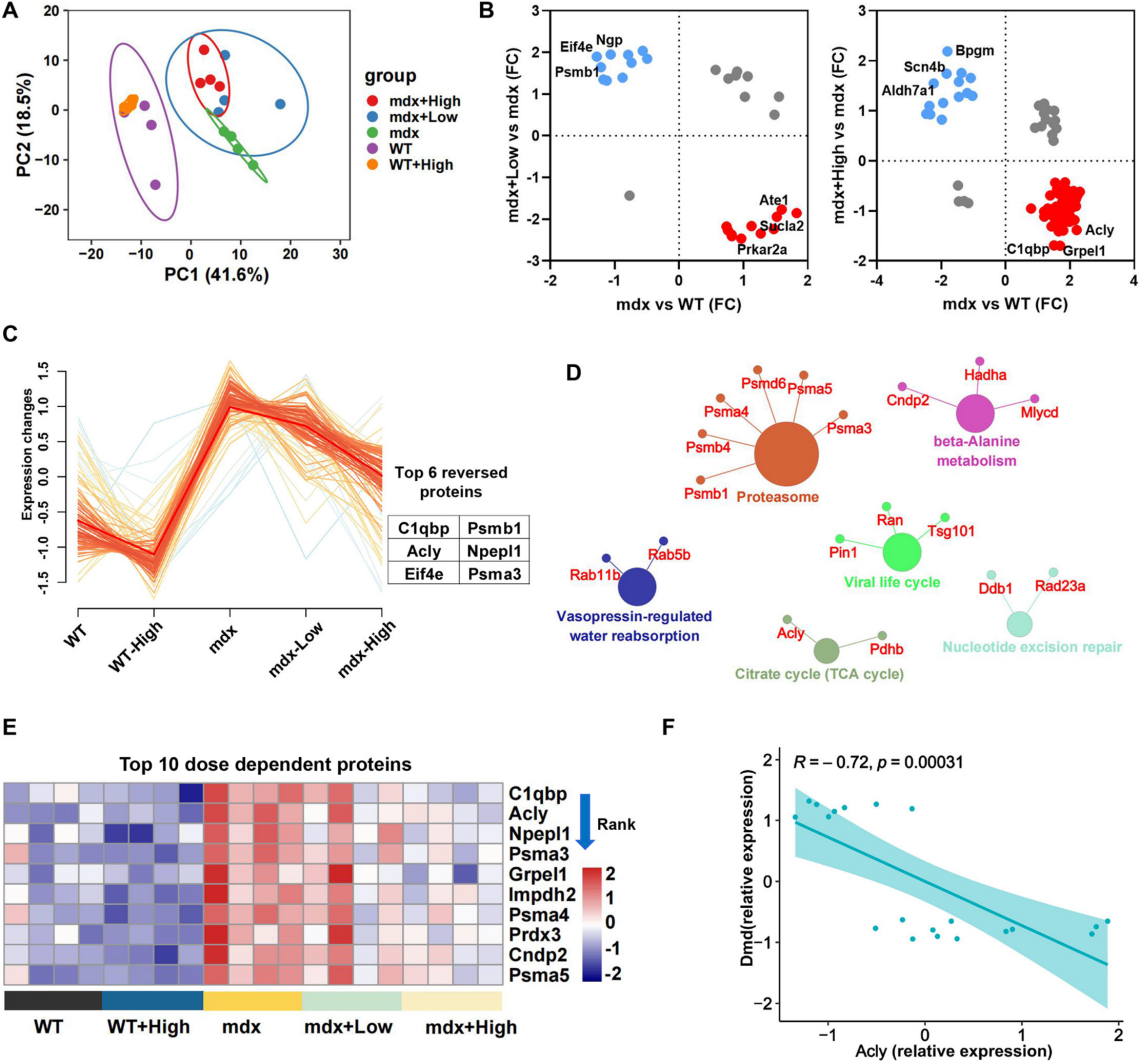
Proteomics analysis of gastrocnemius in mdx mice after ARC‐18 treatment. (A) The principal component analysis (PCA) of all the quantified proteins in experimental mice. (B) Two‐dimensional comparison of differentially expressed proteins in different compared groups. Blue represents down‐regulation in the mdx mice and up‐regulation after ARC‐18 administration, red represents up‐regulation in the mdx mice and down‐regulation after administration. (C) The cluster analysis to found a protein module with reversed expression, and the top 6 reversed proteins were showed in the right, followed by KEGG pathway analysis (D). Different colours represent different signalling pathways, and the enriched proteins in the pathways have also been shown. (E) The dose dependent analysis of all the differentially expressed proteins after ARC‐18 treatment, and the top 10 dose dependent proteins were listed according to fold changed rank. (F) Correlation analysis of the expression of Acly and Dmd.

In order to explore the mechanisms of ARC‐18 therapeutic effect, the proteomic analysis of gastrocnemius was conducted in mdx mice. The differentially expressed (DE) analysis showed a total of 1018 DE proteins were found in the compare group of mdx versus WT mice (Table S2). The low dose of ARC‐18 treated mice induced 92 DE proteins (subsequently referred to as ‘differential proteins’) compared with mdx vehicle mice (Table S3), and a total of 169 DE proteins were found in high‐dose ARC‐18 treated mice (Table S4). Unsupervized principal component analysis (PCA) for the proteome showed that the protein expression pattern was obviously discriminated between mdx treated or untreated mice and WT treated or untreated mice. PCA cycle also showed a discrimination of protein expression between high dose ARC‐18–treated mdx mice and vehicle‐treated mdx mice, while the high dose ARC‐18 treated WT mice showed a similar expression pattern with WT vehicle mice (Figure 4A). The PCA analysis briefly proved the therapeutic effect of ARC‐18 treatment in mdx mice.

Compared to the DE proteins derived from comparison groups of mdx versus WT, low doses of ARC‐18 treated mdx mice (mdx + low) versus mdx and high doses of ARC‐18 treated mdx mice (mdx + high) versus mdx, we showed the top 6 reversal proteins after ARC‐18 treatment, such as neutrophilic granule protein (Ngp), eukaryotic translation initiation factor 4E (Eif4e), proteasome subunit beta type‐1 (Psmb1), arginyl‐tRNA‐protein transferase 1 (Ate1), succinate‐CoA ligase [ADP‐forming] subunit beta, mitochondrial (Sucla2) and cAMP‐dependent protein kinase type II‐alpha regulatory subunit (Prkar2a) in the compared group of mdx + low versus mdx. The top 6 reversal proteins of bisphosphoglycerate mutase (Bpgm), sodium channel subunit beta‐4 (Scn4b), alpha‐aminoadipic semialdehyde dehydrogenase (Aldh7a1), ATP‐citrate synthase (ACLY), complement component 1 Q subcomponent‐binding protein, mitochondrial (C1qbp) and GrpE protein homologue 1, mitochondrial (Grpel1) were found in the compared group of mdx + high versus mdx (Figure 4B). In order to find out the protein module that showed reversal expression, the cluster analysis was performed on a total of 235 DE proteins after ARC‐8 treatment (Table S5). Most of the DE proteins enriched in a cluster of up‐regulation in mdx mice followed by down‐regulation in ARC‐18 treated mdx mice, and the top 6 reversal proteins were C1qbp, ACLY, Eif4e, Psmb1, probable aminopeptidase NPEPL1 (Npepl1) and proteasome subunit beta type‐1 (Psmb3) (Figure 4C). Then, the KEGG pathway analyzed this cluster and found that the enriched pathway contains proteasome, beta‐alanine metabolism, viral life cycle, citrate cycle (TCA cycle), etc., and the top reversal protein of ACLY also focused on the pathway of TCA cycle (Figure 4D).

The Venny analysis found a total of 116 DE proteins overlapped in compared groups of mdx versus WT and ARC‐18 treated mdx versus mdx (Table S6). In order to find the crucial protein after ARC‐18 treatment, the dose‐dependent analysis filtered out 64 DE proteins with dose‐dependent changes, and 53 of these DE proteins were also derived from cluster 1 (Table S7). Then, we showed the top 10 dose‐dependent DE proteins in Figure 4E; ACLY was also the protein with a high differential rank. Moreover, the correlation analysis found that the expression of ACLY was significantly negatively correlated with the expression of dystrophin (Dmd) (Figure 4F). A previous study proved that the increased ACLY led to fibrogenesis in chronic kidney disease (CKD) [21]. The proteomic analysis indicated that ARC‐18 modified the abnormal expression of proteins involved in proteolysis, inflammation, oxidative stress, etc., and the down‐regulation of ACLY may suppress the fibrosis in mdx mice.

### 3.5 ARC‐18 Suppressed the Fibrogenesis by Inhibiting the ACLY‐Mediated Smad2/3 Acetylation

**FIGURE 5:**
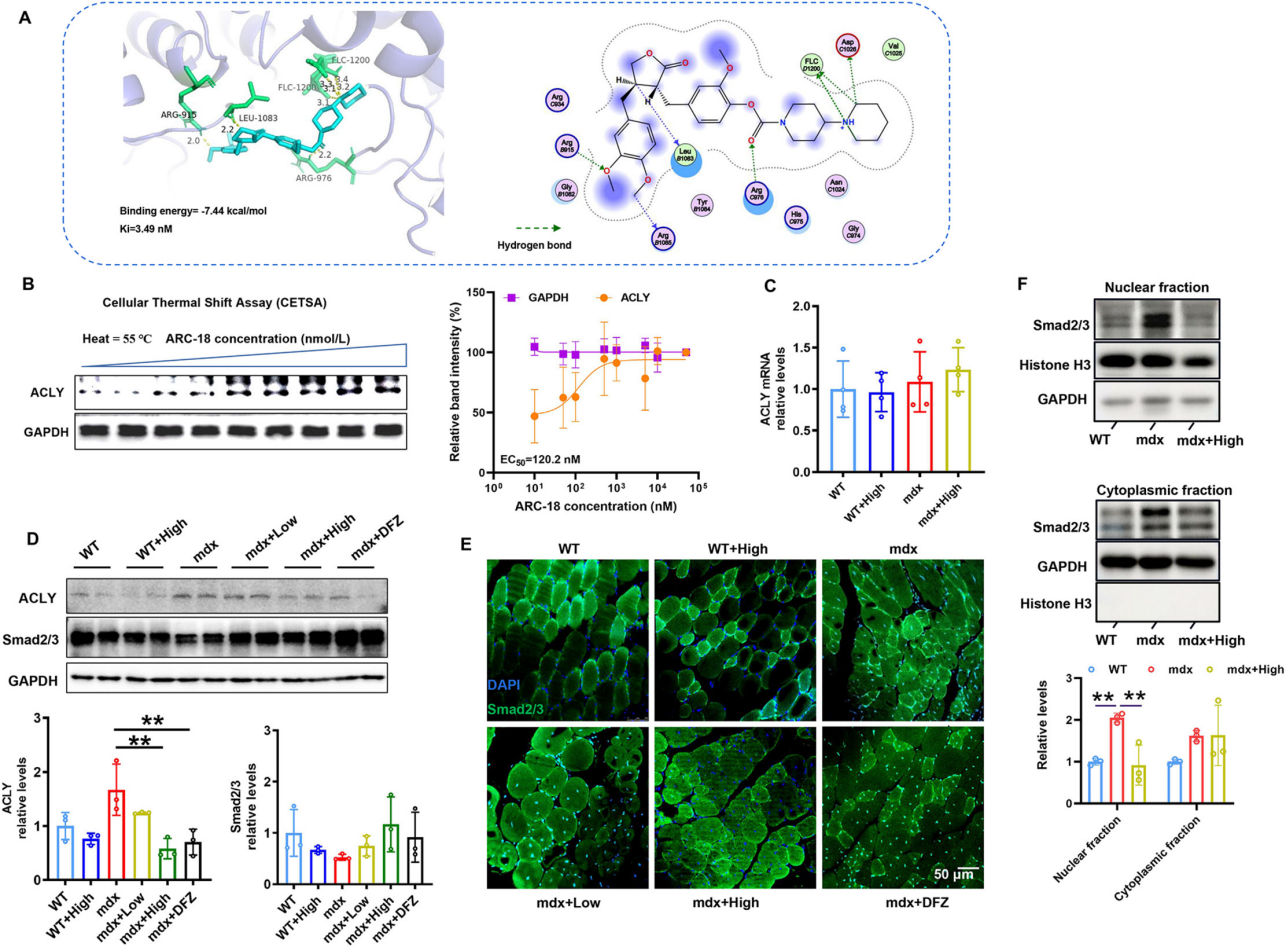
ARC‐18 directly targeted ACLY and suppressed nuclear location of Smad2/3 in gastrocnemius of mdx mice. The molecular docking (AutoDock Vina) and Cellular Thermal Shift Assay (CETSA) were used to confirm ARC‐18 target ACLY, then the expression of ACLY and Smad2/3 location were detected after ARC‐18 treatment in mdx mice. (A) The illustration diagram of ARC‐18 and ACLY protein docking demonstrated the specific docking sites as well as the locations of hydrogen bonding interactions. (B) Western blot showed the thermal stability of ACLY protein at different concentrations of ARC‐18 at 55°C, and calculated the corresponding EC_50_ concentration. (C) The mRNA level of ACLY after high dose of ARC‐18 treatment. (D) Western blot analysis of ACLY and Smad2/3. (E) and (F) Immunofluorescence or western blot analyzed expression of Smad2/3 in the cytoplasm or nucleus after ARC‐18 treatment. Data were shown as mean ± SD. *, *p* < 0.05, **, *p* < 0.01, *n* = 3 for each group.

In this study, proteomics analysis revealed that ACLY may be the therapeutic target of ARC‐18 in mdx mice (Figure 4). Together, these studies indicate that ARC‐18 may inhibit TGF β1 signalling by targeting ACLY to reduce fibrosis in mdx mice. To confirm whether ARC‐18 can directly interact with ACLY, the computer molecular docking (AutoDock Vina) and cellular thermal shift assay (CETSA) were performed. As shown in Figure 5A, ARC‐18 can form multiple hydrogen bonding sites with the ACLY protein. Moreover, ARC‐18 also obviously improved the thermal stability of ACLY protein as reflected by western blots, and the half effective dose of 55°C thermal stability was 120.2 nM (Figure 5B). These results proved that ACLY bound with ARC‐18 directly. Due to the decreased expression of ACLY found after ARC‐18 treatment in proteomics analysis, the corresponding mRNA and protein of ACLY were measured in ARC‐18 treated mdx mice. The mRNA of ACLY showed no difference between mdx mice and high dose of ARC‐18 treated mdx mice (Figure 5C), while the protein expression of ACLY was significantly decreased after ARC‐18 treatment in mdx mice (Figure 5D). This result indicated that ARC‐18 may promote ACLY protein degradation. Despite no significant difference in the protein level of Smad2/3, the level of Smad2/3 in the nucleus was obviously reduced after ARC‐18 treatment (Figure 5E,F). These results indicated that ARC‐18 can inhibit ACLY/Smad2/3 signalling in mdx mice.

**FIGURE 6:**
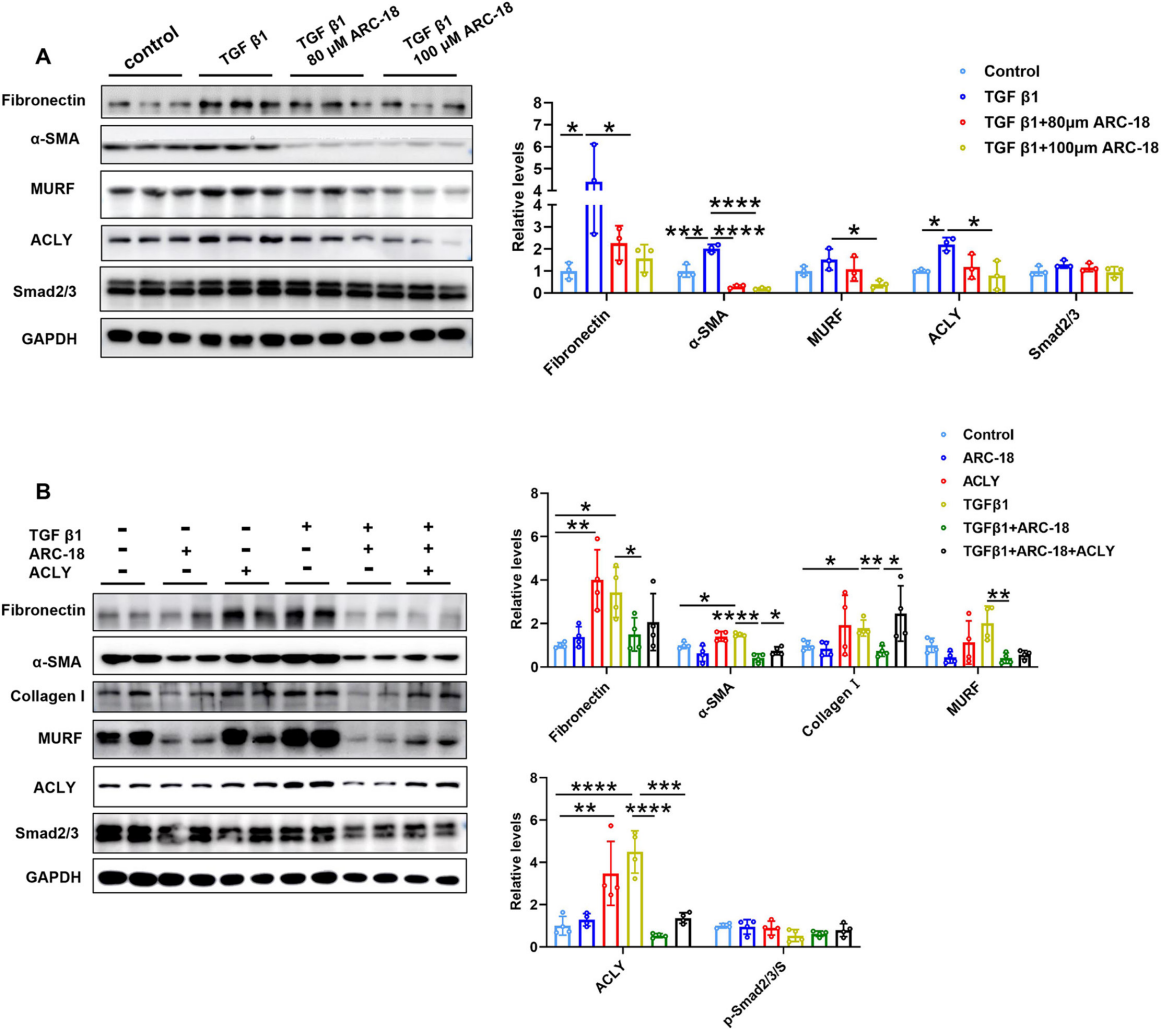
ARC‐18 suppressed fibrosis through ACLY‐mediated TGF‐β1 signalling. (A) Western blot and quantification of Fibronectin, α‐SMA, MURF, ACLY and Smad2/3 in C2C12 cell treat with 80 μM or 100 μM ARC‐18 after 5 ng/mL TGF‐β1 pretreatment. (B) After ACLY over expression, western blots and quantification of Fibronectin, α‐SMA, Collagen I, MURF, ACLY and Smad2/3 in C2C12 cell treat with 100 μM ARC‐18 and 5 ng/mL TGF β1. Data were shown as mean ± SD. *, *p* < 0.05, **, *p* < 0.01, ***, *p* < 0.001, ****, *p* < 0.0001. *n* = 4 for each group.

To investigate whether ARC‐18 exerted antifibrosis function by targeting ACLY, the in vitro experiments were performed in mouse myoblasts (C2C12 cell). In order to find the optimal concentration and incubation time of TGF β1 in C2C12 cells, 5 ng/mL or 10 ng/mL TGF β1 was treated to C2C12 cells for 1, 3, 6, 12, or 24 h. We found that 5 ng/mL TGF β1 treated C2C12 cells for 12 h significantly increased the expression of α‐SMA, MURF and ACLY (Supplementary Figure 5A). The cell viability of ARC‐18 in C2C12 cells was assayed by cell counting kit‐8 (CCK8), and the IC_50_ of ARC‐18 was 296.48 μM (Supplementary Figure 5B). Thus, we selected 80 μM, 100 μM ARC‐18 to treat 5 ng/mL TGF β1‐preincubated C2C12 cells for 12 h and found that the expression of Fibronectin, α‐SMA, MURF and ACLY was significantly decreased after 100 μM ARC‐18 treatment (Figure 6A). In addition, the overexpression of ACLY in C2C12 cells attenuated the improvement effects of ARC‐18 on the suppression of fibrosis, reflected by the significantly increased expression of α‐SMA and Collagen I (Figure 6B). These results confirmed that ARC‐18 suppressed the expression of fibrosis‐related proteins induced by TGF β1 was mediated by targeting ACLY to decrease its expression.

**FIGURE 7:**
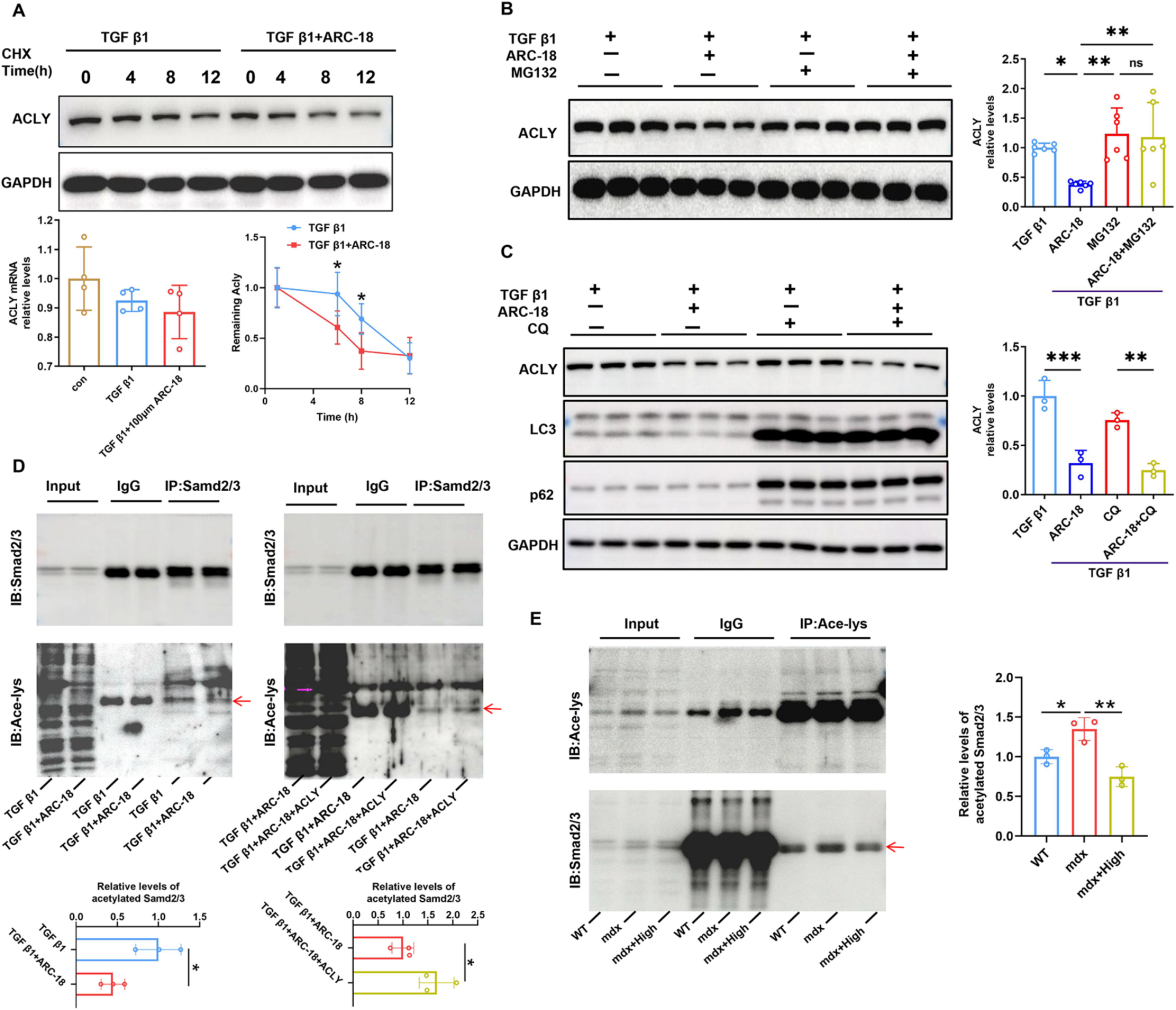
ARC‐18 promoted the proteasome degradation pathway of ACLY and inhibited ACLY‐mediated Smad2/3 acetylation. Western blot analysis of ACLY expression at 0, 4, 6 and 8 h in TGF β1 preincubated C2C12 cell after ARC‐18 or cycloheximide (CHX) treatment (A), or in MG132 treatment C2C12 cell (B), and expression of ACLY, p62 and LC3 in CQ treatment C2C12 cell (C). (E) The immunoprecipitation analysis of acetylation levels of Smad2/3 in TGF β1 preincubated C2C12 cell after ARC‐18 or over expression ACLY treatment and (F) in gastrocnemius of ARC‐18 treated mdx mice. Data were shown as mean ± SD.*, *p* < 0.05, **, *p* < 0.01, ***, *p* < 0.001. (A) *n* = 4, (B) *n* = 6, (C–E) *n* = 3 per group.

**FIGURE 8:**
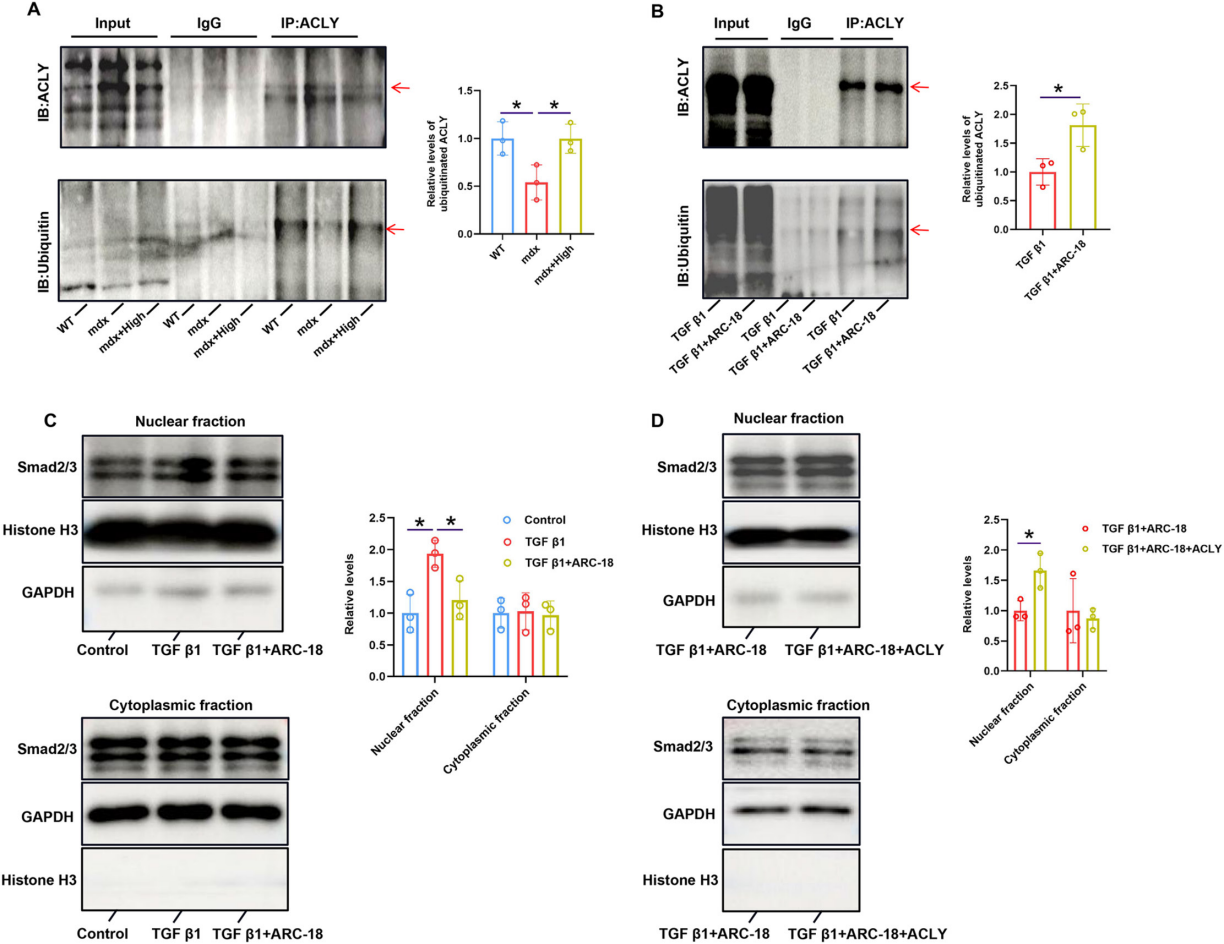
ARC‐18 treatment suppressed nuclear translocation of Smad2/3 and promoted ubiquitination levels of ACLY. The immunocoprecipitation analysis of ubiquitination levels of ACLY in mdx mice (A), or TGF β1‐preincubated C2C12 cell (B) after ARC‐18 treatment. (C) and (D) Western blots analysis expression of Smad2/3 in nuclear fraction or cytoplasmic fraction of TGF β1‐preincubated C2C12 cell after ARC‐18 administration. Data were shown as mean ± SD..*, *p* < 0.05, vs. con group. *n* = 3 for each group.

To clarify how ARC‐18 reduced the expression level of ACLY protein and suppressed TGF β1 signalling, the protein degradation experiment and detection of Smad2/3 acetylation were performed in C2C12 cells treated with ARC‐18 in combination with TGF β1. Consistent with the results in animals, ARC‐18 treatment of C2C12 cells did not cause significant changes in ACLY mRNA levels. After cycloheximide (CHX) administration to inhibit protein synthesis, ARC‐18 promoted the ACLY degradation in TGF β1‐preincubated C2C12 cells as reflected by a significant decrease in ACLY expression compared with untreated control cells (Figure 7A). The proteasome inhibitor MG132 combined with ARC‐18 significantly suppressed ACLY protein degradation induced by ARC‐18 administration alone (Figure 7B), while the autophagy inhibitor chloroquine (CQ) combined with ARC‐18 showed no different expression of ACLY compared with ARC‐18 treated alone (Figure 7C). Moreover, the immunoprecipitation experiment showed that ARC‐18 administration promoted ubiquitination of ACLY in mdx mice or TGF β1‐preincubated C2C12 cells (Figure 8A,B). These results confirmed that ARC‐18 promoted ACLY protein degradation through the proteasome pathway.

In addition, the in vitro experiment also proved that ARC‐18 suppressed the nuclear localization of Smad2/3, while overexpressed ACLY reversed this effect, and both treatments did not influence Smad2/3 levels in the cytoplasmic fraction(Figure 8C,D). A previous study reported that Smad2/3 can be acetylated and this acetylation controls its nuclear translocation and transcriptional activity [22, 23]. ACLY can enhance the acetylation of Smad3 by increasing the level of acetyl‐CoA, thus promoting the nuclear localization and activity of Smad3 [24]. In this study, ARC‐18 obviously reduced ACLY‐mediated acetylation of Smad2/3 in TGF β1‐preincubated C2C12 cells (Figure 7D) and in mdx mice (Figure 7E). All these results proved that ARC‐18 suppressed fibrogenesis by inhibiting the ACLY‐mediated Smad2/3 acetylation.

Cardiac fibrosis was reduced in mdx mice after ARC‐18 treatment (Supplementary Figure 2B), we assessed ACLY expression in the hearts of mdx mice and found that ARC‐18 treatment significantly reduced ACLY protein levels compared to untreated controls (−42.7%, *p* < 0.05) (Supplementary Figure 6A). Proteomic analysis revealed that ARC‐18 treatment modulates the expression of several electron transport chain proteins (such as Mtatp8, Ndufb7) (Supplementary Figure 6B). Similarly, muscle fibre type induced by ARC‐18 treatment in mdx mice transitions from slow‐oxidative fibres toward faster, more oxidative‐glycolytic fibres, as evidenced by a significant decrease in Myh7 expression (*p* < 0.0001) and an increase in Myh1 expression (*p* < 0.05) in muscle (Supplementary Figure 6C). Additionally, analysis of the mRNA dataset (GSE109178) from the muscles of DMD patients revealed a significant upregulation in the expression level of ACLY (Supplementary Figure 7). This suggested that ACLY may represent a promising therapeutic target for DMD. Furthermore, ARC‐18 may also provide potential benefits for DMD patients.

## 4 Discussion

As a rare and fatal muscle disease, DMD is characterized by muscle atrophy and fibrosis, which is the major cause of death [1]. Although a number of therapies have already been approved, the development of effective therapies for DMD is still ongoing [25]. Arctigenin has anti‐inflammatory activity in the tumour microenvironment [14] and reduces myofibroblast activities during oral submucous fibrosis [13], etc. In this study, based on the molecular structure of Arctigenin, we designed and synthesized ARC‐18 and found that ARC‐18 treatment (a) ameliorated the motor system dysfunction, (b) preserved the integrity of the gastrocnemius and triceps, (c) reduced the levels of CK in serum and inflammatory factors in the gastrocnemius, (d) attenuated the development of the abnormal protein profile in the gastrocnemius, (e) reduced the fibrosis areas in the gastrocnemius and diaphragmatic muscle and (f) inhibited the phenotypic conversion of myoblasts into fibroblasts, which suggests that ARC‐18 is a potential drug for DMD.

Inflammation and fibrosis are important factors leading to disease deterioration and eventual death in DMD patients. TGF β1 played a key role in fibrosis during chronic injury [26], and induced the proliferation and activation of interstitial fibroblasts after epithelial cells were injured [27]. The previous research proved that TGF β1 increased in the muscles of DMD patients, and inhibiting TGF β1 can alleviate fibrosis in DMD [28]. TGF β1 induced skeletal muscle atrophy requires the activation of the canonical (TGF β1/Smad2/3) signalling pathway [29]. Moreover, knockdown of ACLY inhibits TGF β1‐induced kidney fibrosis [21], acetyl‐CoA can promote the activation of Smad2/3 [24, 30] and ACLY is one of the main enzymes that enhance the acyl‐CoA level [31]. All these indicated ACLY may be an important downstream regulator of the TGF β1 signalling pathway. Due to the potent profibrotic effects of TGF β1 being well confirmed [32, 33], the anti‐TGF‐β therapy can be an effective approach in DMD, which has been mentioned in DMD model mice [8]. In this study, ARC‐18 treatment significantly decreased TNF‐α, INF‐γ and TGF β1, with the mechanisms of activating the nuclear factor erythroid‐2‐related factor 2/heme oxygenase‐1 signalling pathway (Supplementary Figure 2B), which have been proved in the study of Arctigenin [12].

The marker proteins of myogenic differentiation and proliferation, collagen hyperplasia and fibrosis were significantly increased in the gastrocnemius of mdx mice. These results indicated that the phenotypic conversion of myoblasts into myofibroblasts may be the crucial pathogenic factors in dystrophin‐deficient mice. A previous study also found the superfluous abnormal phenotypic conversion of myoblasts into myofibroblasts [34], and the cell‐autonomous abnormalities in proliferating myoblasts in DMD model mice [35], TGF β1 can induce the phenotypic conversion of myoblasts into myofibroblasts [36]. However, the ARC‐18 treatment reversed this defect, improved myocellular quality and suppressed fibrosis in mdx mice. The immune‐histochemistry or ‐fluorescence data showed that ARC‐18 treatment increased the expression level of MHC and eMyHC, which also indicated that ARC‐18 maintained the normal phenotypic conversion of myoblasts in mdx mice.

Although TGF β1 induced fibrosis is a classical pathway, it also requires other inducements to maintain this process, such as increased acetylation of Smad2/3 to promote signal transduction [37]. Thus, we performed proteomics analysis to explore the possible mechanism of ARC‐18 treatment in mdx mice. From bioinformatic analysis, we found ACLY not only was the top reversed protein but also the top dose‐dependent changed protein, and ACLY enriched in the TCA cycle pathway. Moreover, the expression of ACLY has a significantly negative correlation with the expression of Dmd (Dystrophin). It has been reported that ACLY inhibition suppressed TGF β1 induced ECM production in fibroblasts [21], pharmacological inhibition of ACLY ameliorates fibrosis in nonalcoholic steatohepatitis (NASH) [38]. Moreover, ACLY is reported to increase the level of acetyl‐CoA to promote acetylation of Smad2/3, and then enhance Smad2/3 nuclear location and activation [24, 30]. All these data indicate that ACLY may be a possible target for ARC‐18 treatment in mdx mice.

In addition, restoring the missing antimuscular dystrophy protein (DMD) and improving muscle mass are effective approaches to treat DMD, such as gene therapy to compensate for DMD protein function [39], compounds that inhibit myostatin to compensate for muscle tissue loss [40]. However, these treatment strategies have also side effects in clinical trials, such as transient renal failure and elevation of liver enzymes [41]. ARC‐18 treatment maintained the muscular quantity reflected by increasing expression of dystrophin, decreasing Even blue staining, significant reduction in CK activity and retention of expression of muscle structural proteins and normal phenotypic conversion of myogenic cells. The inhibitory effect of fibrosis of ARC‐18 obviously ameliorated the skeletal muscle degeneration, as shown in the reduction of the inflammation area and collagen fibre in mdx mice. ARC‐18 exerted notable therapeutic effects in mdx mice, including inhibition of collagen fibrillogenesis and reduction of pro‐inflammatory cytokines such as TNF‐α and TGF‐β1. In some aspects, ARC‐18 showed a trend toward improved efficacy compared to the positive control drug DFZ. However, this superiority was not consistently observed across all functional outcomes, particularly in motor performance, where both compounds showed comparable benefits in several tests.

This study has several limitations that should be acknowledged. First, the experiments were conducted using the mdx mouse model, which, although widely used in DMD research, does not fully replicate the progressive severity of the human disease, especially with respect to cardiac and respiratory involvement. Second, ARC‐18 treatment was initiated prophylactically in young mice before significant disease progression, which may not fully reflect the clinical context in which DMD patients typically begin therapy. Future studies will be needed to assess the therapeutic efficacy of ARC‐18 in a treatment rather than prevention paradigm. Although the data suggest that ARC‐18 may inhibit the phenotypic conversion of myoblasts into fibroblasts in mdx mice, direct detection of fibroblast markers and lineage tracing would be required to conclusively confirm transdifferentiation events. Third, while ARC‐18 demonstrated beneficial effects in short‐term administration, we did not evaluate long‐term outcomes or potential adverse effects, which are essential for clinical translation. Finally, although the current dosing strategy was effective in mice, the pharmacokinetics, toxicity profile and optimal dosage of ARC‐18 in humans remain unknown and require further investigation through preclinical and clinical studies.

## 5 Conclusions

We have identified a new antifibrotic and antiatrophic small‐molecule, ARC‐18, which can suppress fibrosis and prevent chronic inflammation, motor dysfunction and pathological deterioration. Further studies show that ARC‐18 inhibits collagen fibrohyperplasia by suppressing ACLY‐mediated Smad2/3 acetylation. This multifunctional small‐molecule agent can be a great promise for future clinical treatment of DMD.

## Conflicts of Interest

The authors declare no conflicts of interest.

## Supporting information


**Data S1:** Supporting Information.

**Supplementary Figure 1:** Information on ARC‐18 and weight of mice after treatment.(A) Structural formula, molecular weight and other chemical information of ARC‐18. (B) Weight changes in mice over the course of 2 months of ARC‐18 treatment. Data were shown as mean ± SD ****, *p* < 0.0001, mdx mice vs. WT mice. *n* = 10 for each group.

**Supplementary Figure 2:** The detection of muscle weight, collagen fibres and Nrf2/HO‐1 signal pathway after ARC‐18 treatment in mdx mice. (A) Wet weight statistics of gastrocnemius, triceps and heart. *n* = 9–10 for each group. (B) Masson Stain for detection of collagen fibres in heart of ARC‐18 treated mdx mice. (C) Western blots analysis of Nrf2 and HO‐1 in gastrocnemius of mdx mice after ARC‐18 administration. *n* = 4 for each group. Data were shown as mean ± SD. *, *p* < 0.05, **, *p* < 0.01. ****, *p* < 0.0001.

**Supplementary Figure 3:** ARC‐18 treatment reduced creatine kinase activity and inflammation levels. (A) Blood biochemical analysis of creatine kinase activity, glucose (GLU), alanine aminotransferase (ALT), aspartate aminotransferase (AST), creatinine and blood urea nitrogen (BUN). (B) Elisa analyzed inflammatory of IL‐1β, TNF‐α and TGF β1 in gastrocnemius after ARC‐18 treatment. Data were shown as mean ± SD. *, *p* < 0.05, **, *p* < 0.01, ***, *p* < 0.001, ****, *p* < 0.0001. *n* = 5–6 for each group.

**Supplementary Figure 4:** ARC‐18 attenuated myoblast differentiation and suppressed fibrosis in mdx mice. Immunofluorescence of the structural protein laminin in gastrocnemius (A) and diaphragm (B). Immunofluorescence and quantification of the regulation of skeletal muscle differentiation protein eMyHC (C). Immunohistochemical and quantification of the collagen fibres composition of Fibronectin in gastrocnemius (D), and the composition of myosin protein MHC in gastrocnemius (E) or in diaphragm (F). Data were shown as mean ± SD. *, *p* < 0.05, **, *p* < 0.01, ***, *p* < 0.001, ****, *p* < 0.0001. *n* = 3 for each group.

**Supplementary Figure 5:** TGF‐β1 treatment induced expression of fibrotic protein in C2C12 cell. (A) Western blot and quantification of Fibronectin, Collagen I, MURF and ACLY in C2C12 cell treat with 5 ng/mL or 10 ng/mL TGF‐β1. Data were shown as mean ± SD. *, *p* < 0.05, **, *p* < 0.01, ***, *p* < 0.001, vs. con group. *n* = 3 for each group. (B) The cell viability of ARC‐18 in C2C12 cell detected by cell counting kit‐8.

**Supplementary Figure 6:** ARC‐18 played a protective role in cardiac muscle function, mitochondrial activity and muscle differentiation. (A) Immunohistochemical and quantification of the ACLY in heart of ARC‐18 treated mdx mice. (B) Heatmap of differential protein expression associated with the mitochondrial electron transport chain in different comparison groups. (C) QPCR analysis of Myh1, Myh4 and Myh7 mRNA expression levels in gastrocnemius after ARC‐18 treatment. Data were shown as mean ± SD. *, *p* < 0.05, **, *p* < 0.01, ****, *p* < 0.0001. *n* = 3 for each group.

**Supplementary Figure 7:** ACLY mRNA expression was increased in DMD patient muscles. Bioinformatic analysis of ACLY mRNA expression in publicly available mRNA expression data (GEO Dataset GSE109178), which includes 17 DMD patient and 6 healthy control quadriceps samples.

**Supplementary Table 1:** The antibody information used in this study.
Supplementary Tables 2–7. The list of differentially expressed (DE) proteins from proteomic analysis.

## Data Availability

All data and materials described in the study are present in the paper or the Supplementary Materials.
