# Supplementary material for: ARC‐18 Improved Motor Performance Through Inhibiting ACLY‐Mediated Smad2/3 Acetylation in a Model of Duchenne Muscular Dystrophy
Source: J Cachexia Sarcopenia Muscle. 2025 Oct 7;16(5):e70081. doi: 10.1002/jcsm.70081 (PMC12501411; doi:10.1002/jcsm.70081)
Supplement: Supplementary file 1 — Data S1: Supporting Information. [file JCSM-16-e70081-s005.docx]

**ARC-18 improved motor performance through inhibiting ACLY-mediated Smad2/3 acetylation in a model of Duchenne muscular dystrophy**

Chongyang Chen^1, 2, 3, #^, Binge Zhang^1, 3, #^, Chao Yang^4, #^, Jing Wang^3^, Ye He^1^, Haitao Yu^1^, Jianjun Liu^3^, Yongmei Xie^4, *^, Xifei Yang^3, *^ and Gong-Ping Liu^1,5, *^

**Supplementary materials**

**Methods**

**Behavioral tests**

**Motor performance**

The methods for evaluating motor performance of rotarod, hanging endurance, climbing pole and grip strength in experiment mice were referenced previous study ^S1^. Briefly, in rotarod test, each animal was placed on a swivel bar fatigue tester with a rotation speed at 30 rpm. The experimenter recorded the running time on the swivel bar (at the bar time) for 5 min, and the cut-off value was set as 300 s. For hanging endurance test, the experimenter places each mouse in the center of a wire grid (line width of ~0.1 cm, spacing, 0.5 cm), and then, tapped the grip to make the mouse grip tight. The grid was slowly inverted to horizontal. The experimenter recorded the time during which the mouse hung onto the grid (grip time), and set 90 s (or longer) as the cut-off value. In climbing pole test, each mouse with head down was gently placed on the top of a homemade wooden pole (~50 cm length, ~1 cm diameter), which was placed vertically on a table-top. The experimenter recorded the time taken for the mouse to descend from the top to the bottom of the platform (climbing time), and the cut-off value was set as 15 s. For grip strength test, the experimenter placed each mouse on the central table of the fingerboard, gently pull the mouse's tail to encourage the mouse to grab the grip plate, and recorded the maximum gripping power. The experiment process for each animal was repeated three times, the largest value was taken as the evaluation value.

**Gait analysis**

The Noldus CatWalk XT system was used for gait analysis of animals to quantitatively evaluate the footsteps and movement of mice after ARC-18 treatment ^S2,3^. Briefly, before the formal test, the mice were given walking training and were allowed to repeatedly walk through the 50 cm-long glass walkways to familiarize themselves with the environment. During the formal test, the mice were put in the aisle entrance, and the mice will walk along the glass aisle. The high-speed Gigabit Ethernet port camera collected the walking data, and the system software Walk Analysator was used for screening and self-analysis. The automatic analysis of footprints could calibrate the wrong footprints.

**Treadmill endurance test**

Treadmill endurance test was usually used for primary phenotypic analysis of muscle performance ^S4^. Briefly, each mouse was trained for 3 days before the formal treadmill experiment, and exercise tolerance was measured by counting the number of electric shocks they received on the uphill treadmill. The treadmill started at a speed of 9 centimeters per second with a 5° inclination. Every 12 min, the speed was gradually increased by 3 cm/sec. If the mouse could not keep up with the treadmill, it was shocked with electricity (0.1 mA). Mice were considered exhausted after accumulating five or more shocks per minute for two minutes in a row on the treadmill, with a maximum running time of 10 min. After the experiment, the total number of shocks were recorded.

**Reverse transcription and real time quantitative PCR**

This experiment was carried out according to manufacturer's instruction (Takara, Dalian, China). The PCR system consisted of 3 mM MgCl2, 0.5 μM forward and reverse primers, 2 μl SYBR Green PCR master mixes, and 2 μl cDNA, and the standards for each gene were prepared using appropriate primers by a conventional PCR. The samples were assayed on a Rotor-Gene 300 Real-time Cycler (Corbett Research, Sydney, Australia). The expression level of the gene was normalized by the housekeeping gene GAPDH. The PCR primers employed in the present study was as follows: ACLY forward and reverse primers, 5′-CAGCCAAGGCAATTTCAGAGC-3′ and 5′-CTCGACGTTTGATTAACTGGTCT-3′; GAPDH forward and reverse primers, 5′-TTCCCGTTCAGCTCTGGG-3′ and 5′-CCCTGCATCCACTGGTGC-3′.

**Western blots**

The gastrocnemius tissue or treated C2C12 cell were ultrasonically lysed with RIPA lysate buffer (or IP lysate buffer) containing protease and phosphatase inhibitor (Thermo Fisher, New Jersey, USA). The cytoplasmic and nucleolar fraction of proteins were extracted and conducted operations in accordance with the instructions of nucleus-cytoplasmic protein-membrane preparation kit (P1201, Applygen). After protein quantification, the sample was mix with loading buffer and boiled at 95 °C for 10 min. Then, samples were loaded on 8%~12% SDS-PAGE and transferred protein onto PVDF membranes followed by blocking with 5% skim milk for 2 h. And then, the membrane was incubated with primary antibody (the antibody information used is shown in Table 1) and secondary antibodies. Finally, the ECL kit (Thermo Fisher, New Jersey, USA) was used to display density of the target protein, and the ImageJ software was used to quantify the relative expression.

**Immunoprecipitation**

The TGF β1, ARC-18 and overexpressed ACLY treated C2C12 cell were homogenized on ice in IP buffer (P0037, Beyotime) and then centrifuged at 12,000×g for 30 min. A total of 200 μg protein was incubated with primary antibodies (Smad2/3 or Ace-lys) overnight, followed by addition of protein G agarose (IP05, Millipore) for 6h (rotating at 4 °C). The agarose beads were washed three times and resuspended in 40 μl of SDS-loading buffer, and then denatured at 95 °C for 10 min. The obtained immunoprecipitant was analyzed by Western blotting.

**Pathological staining**

After fixed with 4% paraformaldehyde, gastrocnemius, diaphragm and heat were dehydrated followed by embedding in paraffin, and cut into 5μm sections. The staining of Hematoxylin and Eosin (H&E), Picro Sirius Red Stain and Masson for pathological examination was performed according to their protocols. For immunohistochemistry and immunofluorescence analysis, the slice was boiled for 10 min in antigen repair solution. Then the slice was washed with PBS and incubated with primary antibody (MHC, eMyhc, Laminin, Fibronectin and Smad2/3) in 0.3% Triton X-100 phosphate buffer normal saline (PBS) at 4 °C overnight. Rabbit-specific HRP/DAB (ABC) Detection IHC Kit was used for immunohistochemistry. Briefly, after incubation with the primary antibody, slice was incubated with biotinylated goat anti-polyvalent for 10 min and washed with PBS, then incubated with streptavidin peroxidase for another 10 min and washed with PBS to staining with DAB for 2-5 min. Finally, slice was stained with hematoxylin, dehydrated with 100-70% gradient ethanol, and cleared with xylene. For immunofluorescence, after incubation with the primary antibody, slice was washed with PBS, followed by incubation with fluorescent secondary antibody and washed with PBS. Finally, slice was stained with DAPI, washed with PBS, and covered with film. **Histological sections were examined under a light microscope and analyzed using Image‑Pro Plus 6.2. We quantitatively evaluated H&E‑stained slides for inflammatory cell infiltration and Masson’s trichrome‑stained slides for collagen deposition in muscle tissue.**

**Proteomics**

The method of proteomics analysis was referred to previous studies ^S5,6^. Briefly, the urea lysis buffer was used to lyse the gastrocnemius tissues and then the obtained protein was digested by trypsin at 37 °C for 14 h. Subsequently, the experimenter labeled the peptide with tandem mass tags (TMT). After dried, the labeled peptide was resolved in 100 μl of 0.1% FA (Formic acid) for further peptide fraction. 15 fractions, which collected from high-performance liquid chromatography (HPLC), were dried and redissolved in 20 µl of 0.1% FA for liquid chromatography (LC)-mass spectrometry (MS)/MS analysis. Using Proteome Discoverer 2.1 software, the obtained data was searched against with UniProt-Mus musculus database. The differential expressed (DE) protein between any compared groups was set p < 0.05.

**Supplementary Figures**

**Supplementary Figure 1. Information on ARC-18 and weight of mice after treatment.**

**Supplementary Figure 2. The detection of muscle weight, collagen fibers and Nrf2/HO-1 signal pathway after ARC-18 treatment in mdx mice.**

**Supplementary Figure 3. ARC-18 treatment reduced creatine kinase activity and inflammation levels.**

**Supplementary Figure 4. ARC-18 attenuated myoblast differentiation and suppressed fibrosis in mdx mice.**

**Supplementary Figure 5. TGF-β1 treatment induced expression of fibrotic protein in C2C12 cell.**

**Supplementary Figure 6. ARC-18 played a protective role in cardiac muscle function, mitochondrial activity, and muscle differentiation.**

**Supplementary Figure 7. ACLY mRNA expression was increased in DMD patient muscles.**


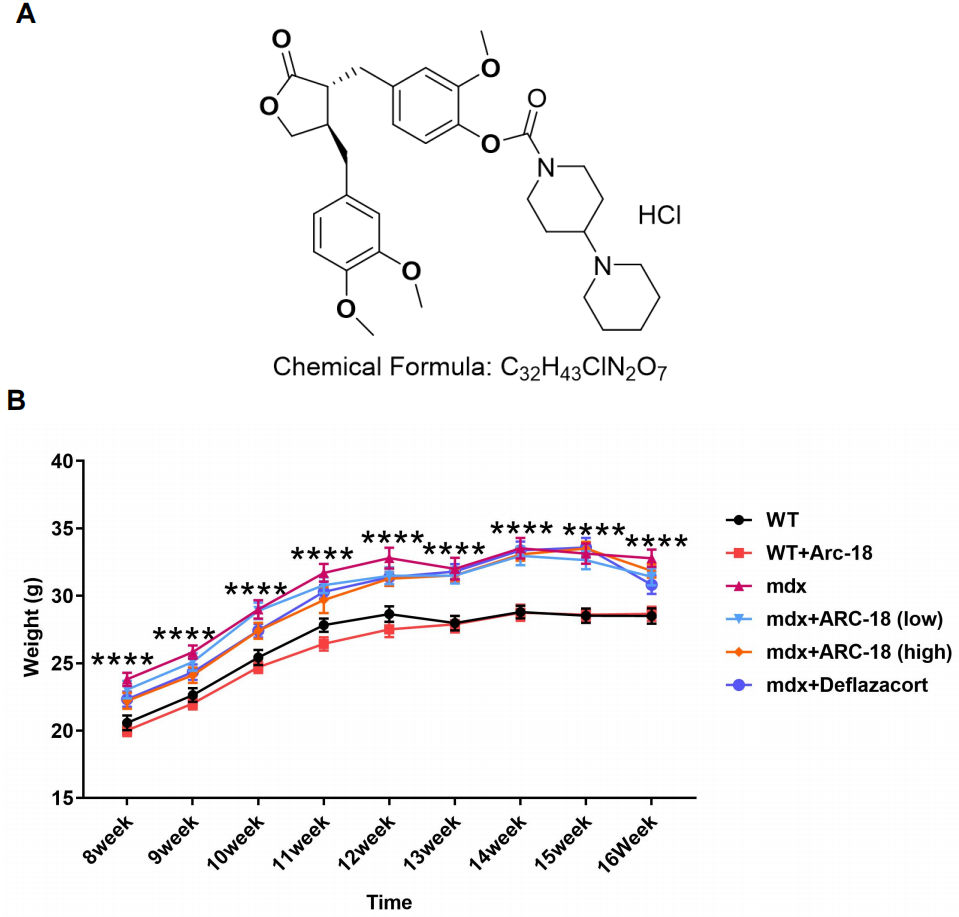


**Supplementary Figure 1. Information on ARC-18 and weight of mice after treatment.**

(A) Structural formula, molecular weight and other chemical information of ARC-18. (B) Weight changes in mice over the course of 2 months of ARC-18 treatment. Data was shown as Mean ± SD. ****, *p* < 0.0001, mdx mice vs WT mice. n = 10 for each group.


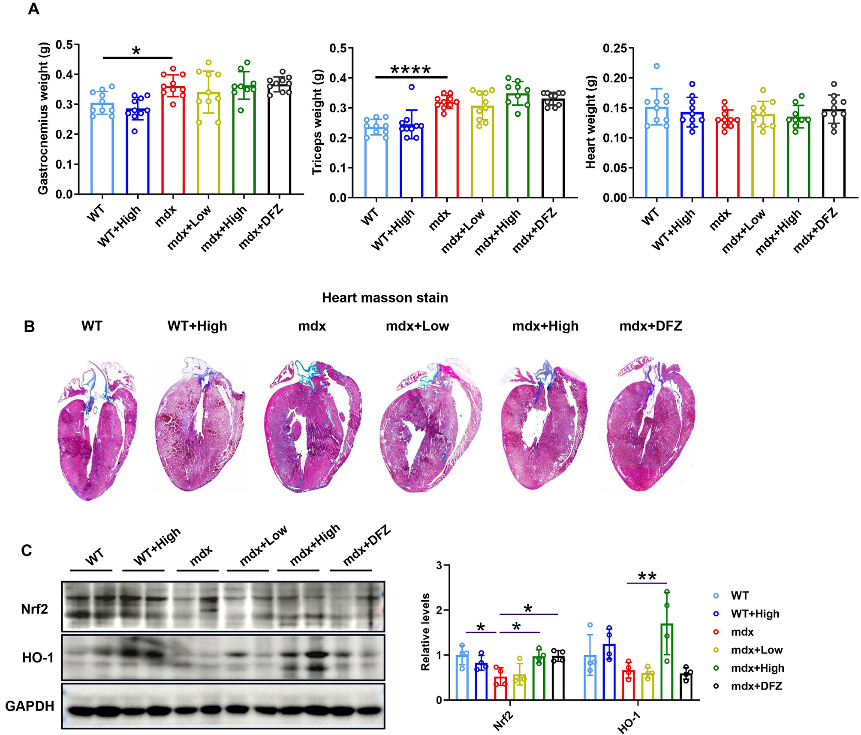


**Supplementary Figure 2. The detection of muscle weight, collagen fibers and Nrf2/HO-1 signal pathway after ARC-18 treatment in mdx mice.**

(A) Wet weight statistics of gastrocnemius, triceps and heart. n = 9-10 for each group. (B) Masson Stain for detection of collagen fibers in heart of ARC-18 treated mdx mice. (C) Western blots analysis of Nrf2 and HO-1 in gastrocnemius of mdx mice after ARC-18 administration. n = 4 for each group. Data was shown as Mean ± SD. *, *p* < 0.05, **, *p* < 0.01. ****, *p* < 0.0001.


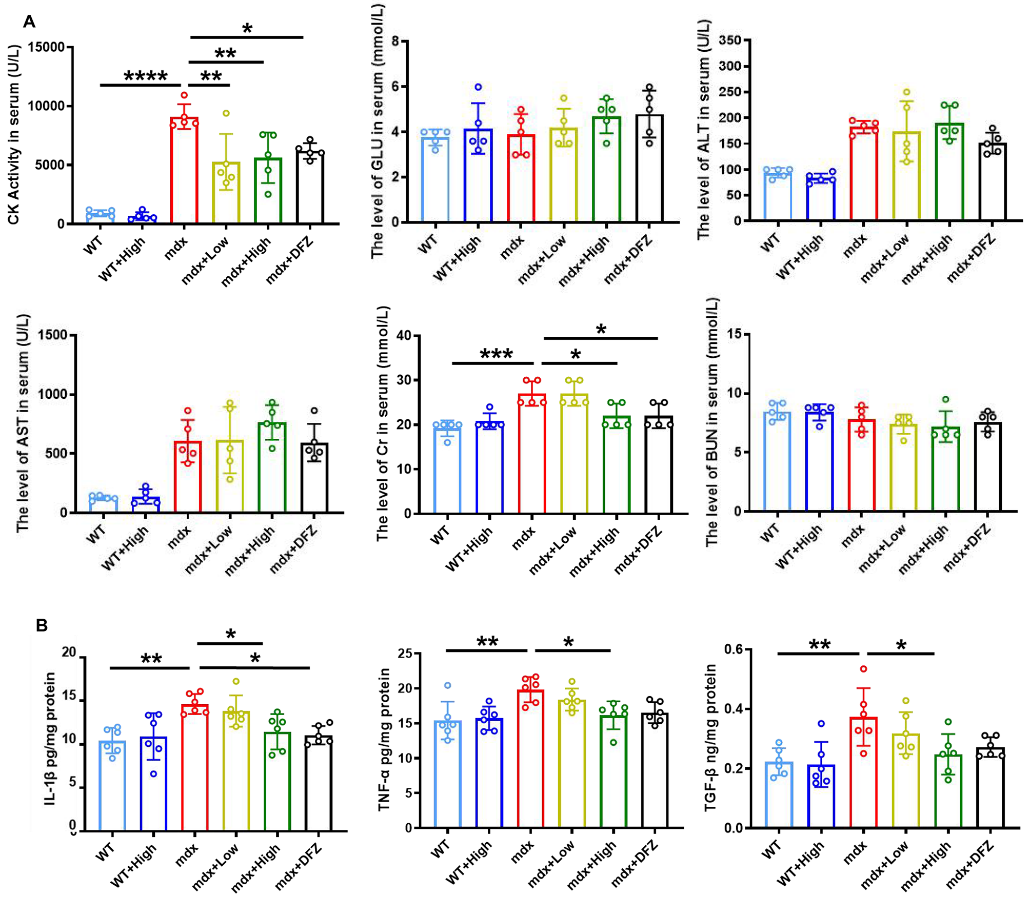


**Supplementary Figure 3. ARC-18 treatment reduced creatine kinase activity and inflammation levels.**

(A) Blood biochemical analysis of creatine kinase activity, glucose (GLU), alanine aminotransferase (ALT), aspartate aminotransferase (AST), creatinine and blood urea nitrogen (BUN). (B) Elisa analyzed inflammatory of IL-1β, TNF-α and TGF β1 in gastrocnemius after ARC-18 treatment. Data was shown as Mean ± SD. *, *p* < 0.05, **, *p* < 0.01, ***, *p* < 0.001, ****, *p* < 0.0001. n = 5-6 for each group.


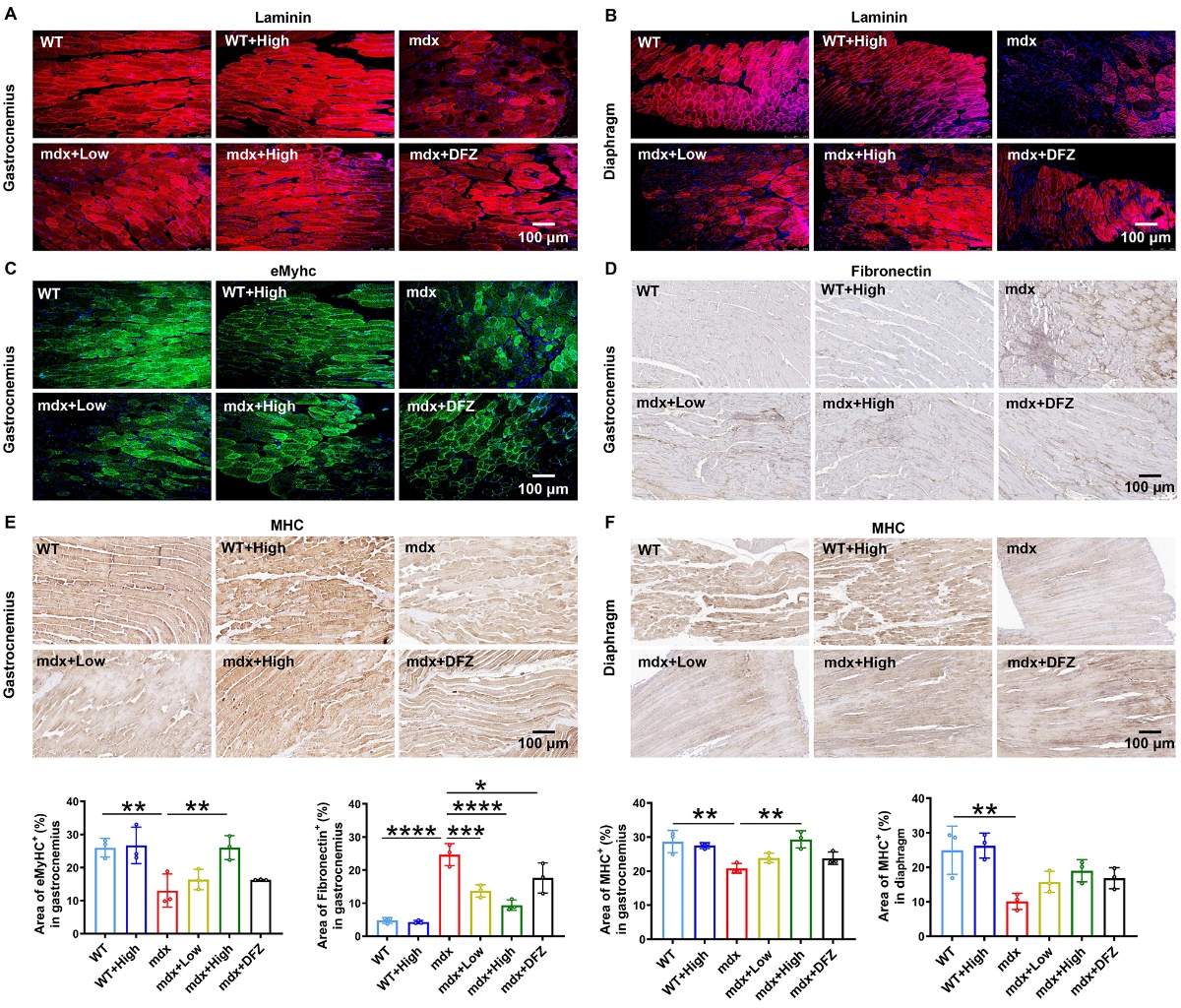


**Supplementary Figure 4. ARC-18 attenuated myoblast differentiation and suppressed fibrosis in mdx mice.**

Immunofluorescence of the structural protein laminin in gastrocnemius (A) and diaphragm (B). Immunofluorescence and quantification of the regulation of skeletal muscle differentiation protein eMyHC (C). Immunohistochemical and quantification of the collagen fibers composition of Fibronectin in gastrocnemius (D), and the composition of myosin protein MHC in gastrocnemius (E) or in diaphragm (F). Data was shown as Mean ± SD. *, *p* < 0.05, **, *p* < 0.01, ***, *p* < 0.001, ****, *p* < 0.0001. n = 3 for each group.


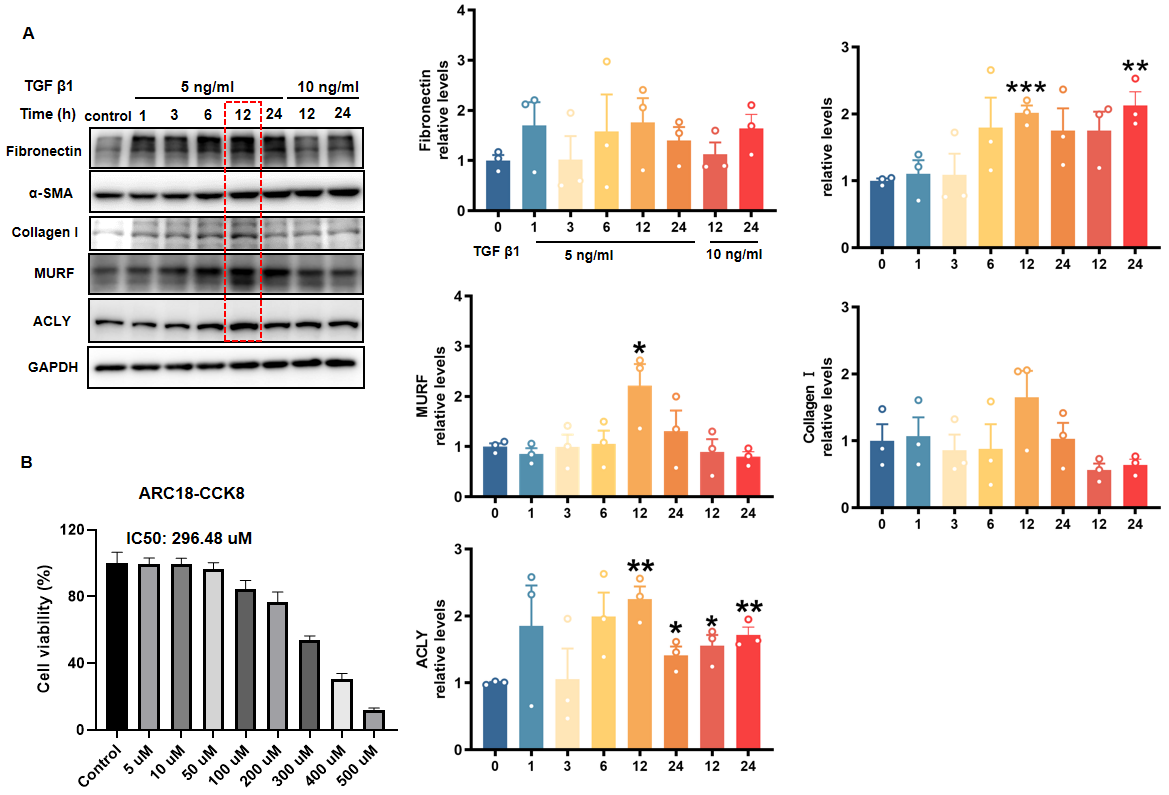


**Supplementary Figure 5. TGF-β1 treatment induced expression of fibrotic protein in C2C12 cell.** (A) Western blot and quantification of Fibronectin, Collagen I, MURF and ACLY in C2C12 cell treat with 5ng/ml or 10 ng/ml TGF-β1. Data was shown as Mean ± SD. *, *p* < 0.05, **, *p* < 0.01, ***, *p* < 0.001, vs con group. n = 3 for each group. (B) The cell viability of ARC-18 in C2C12 cell detected by cell counting kit-8.


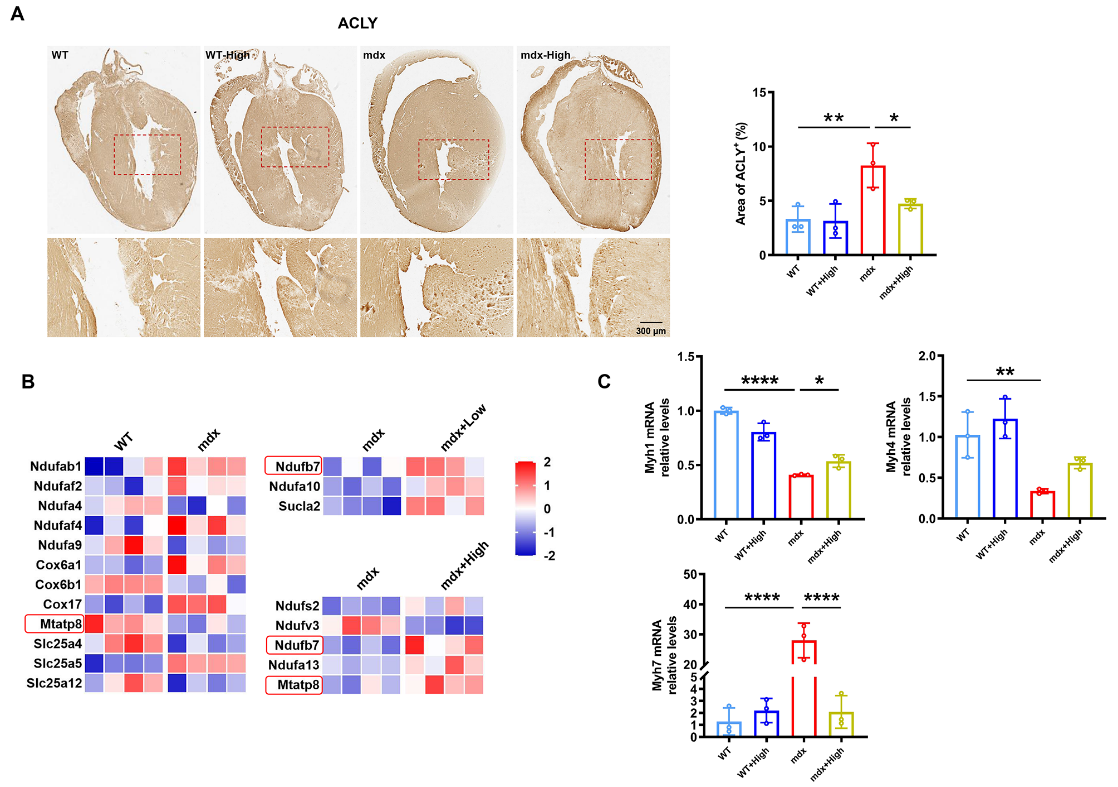


**Supplementary Figure 6. ARC-18 played a protective role in cardiac muscle function, mitochondrial activity, and muscle differentiation.**

**(A) Immunohistochemical and quantification of the ACLY in heart of ARC-18 treated mdx mice. (B) Heatmap of differential protein expression associated with the mitochondrial electron transport chain in different comparison groups. (C) QPCR analysis of Myh1, Myh4 and Myh7 mRNA expression levels in gastrocnemius after ARC-18 treatment. Data was shown as Mean ± SD. *, *p* < 0.05, **, *p* < 0.01, ****, *p* < 0.0001. n = 3 for each group.**


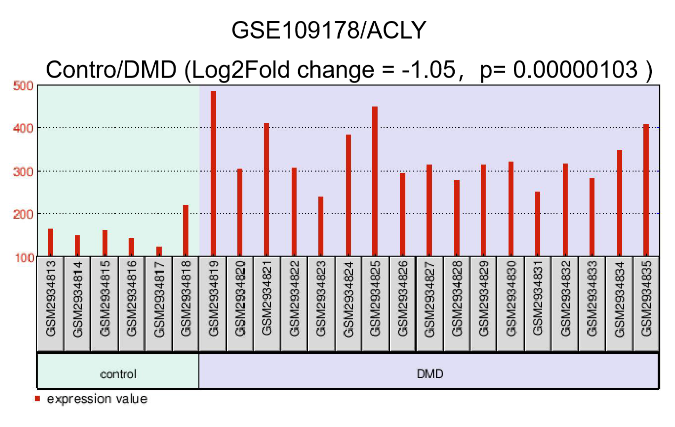


**Supplementary Figure 7. ACLY mRNA expression was increased in DMD patient muscles.**

**Bioinformatic analysis of ACLY mRNA expression in publicly available mRNA expression data (GEO Dataset GSE109178), which includes 17 DMD patient and 6 healthy control quadriceps samples.**

**Supplementary Tables**

**Supplementary Table 1. The information of antibody and chemical reagent were used in this study.**

**Supplementary Table 2-7. The list of differentially expressed (DE) proteins from proteomic analysis.**

**References**

S1. X. Li, C. Chen, X. Zhan, B. Li, Z. Zhang, S. Li, Y. Xie, X. Song, Y. Shen, J. Liu, P. Liu, G.P. Liu, X. Yang, R13 preserves motor performance in SOD1(G93A) mice by improving mitochondrial function, Theranostics 11(15) (2021) 7294-7307.

S2. J.M. Garrick, L.G. Costa, T.B. Cole, J. Marsillach, Evaluating Gait and Locomotion in Rodents with the CatWalk, Curr Protoc 1(8) (2021) e220.

S3. C. Pitzer, B. Kurpiers, A. Eltokhi, Gait performance of adolescent mice assessed by the CatWalk XT depends on age, strain and sex and correlates with speed and body weight, Sci Rep 11(1) (2021) 21372.

S4. B. Castro, S. Kuang, Evaluation of Muscle Performance in Mice by Treadmill Exhaustion Test and Whole-limb Grip Strength Assay, Bio Protoc 7(8) (2017).

S5. C. Chen, C. Yang, J. Wang, X. Huang, H. Yu, S. Li, S. Li, Z. Zhang, J. Liu, X. Yang, G.P. Liu, Melatonin ameliorates cognitive deficits through improving mitophagy in a mouse model of Alzheimer's disease, J Pineal Res 71(4) (2021) e12774.

S6. C. Chen, J. Wang, C. Yang, H. Yu, B. Zhang, X. Yang, B. Xiong, Y. Xie, S. Li, Z. Zhang, F. Zhu, J. Liu, G.P. Liu, X. Yang, Multiomics analysis of human peripheral blood reveals marked molecular profiling changes caused by one night of sleep deprivation, MedComm (2020) 4(3) (2023) e252.
