# Supplementary figures and images for: ARC‐18 Improved Motor Performance Through Inhibiting ACLY‐Mediated Smad2/3 Acetylation in a Model of Duchenne Muscular Dystrophy
Source: J Cachexia Sarcopenia Muscle. 2025 Oct 7;16(5):e70081. doi: 10.1002/jcsm.70081 (PMC12501411; doi:10.1002/jcsm.70081)

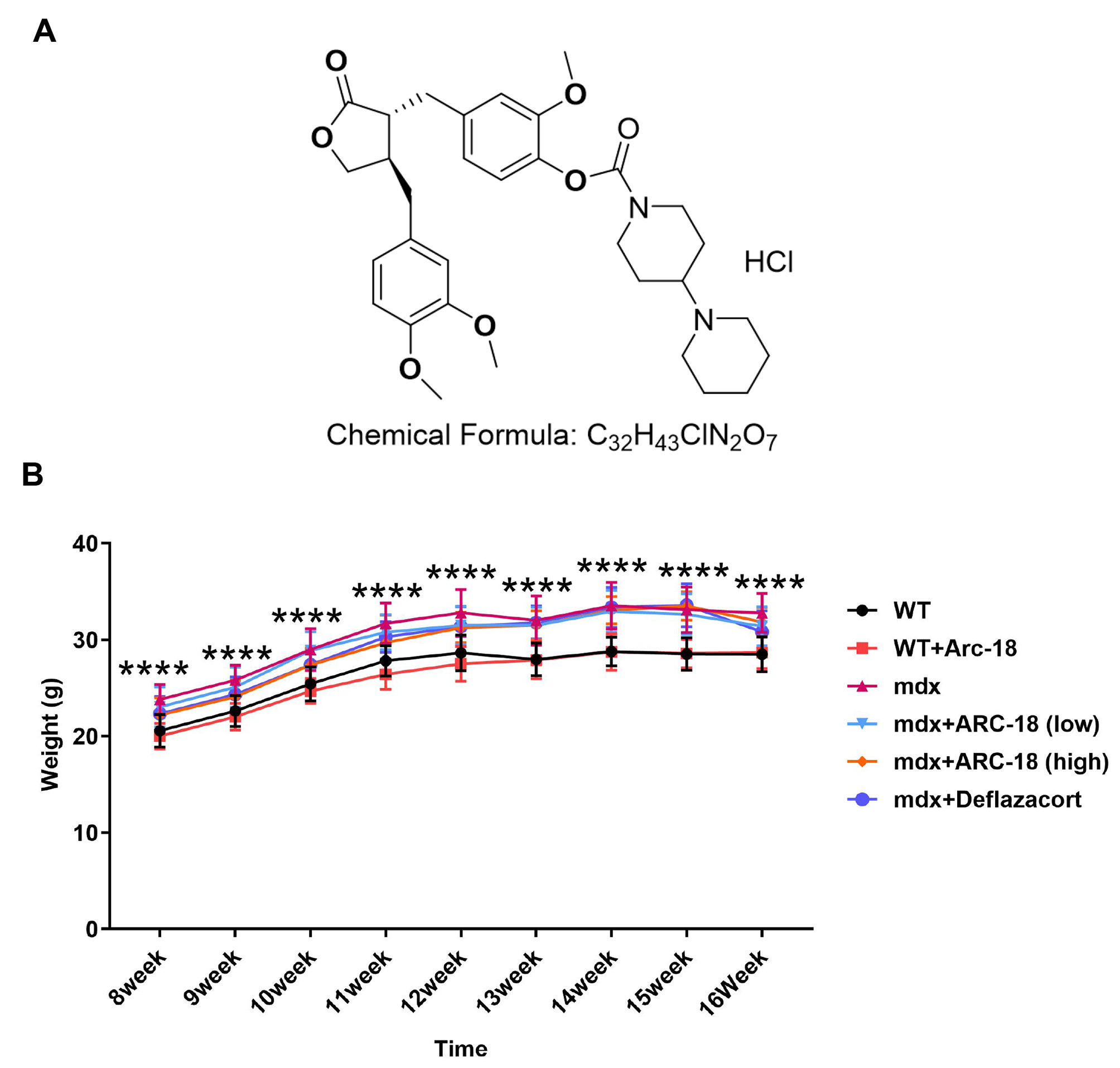

Supplement: Supplementary file 2 — Supplementary Figure 1: Information on ARC‐18 and weight of mice after treatment.(A) Structural formula, molecular weight and other chemical information of ARC‐18. (B) Weight changes in mice over the course of 2 months of ARC‐18 treatment. Data were shown as mean ± SD ****, p < 0.0001, mdx mice vs. WT mice. n = 10 for each group. [file JCSM-16-e70081-s004.tif]

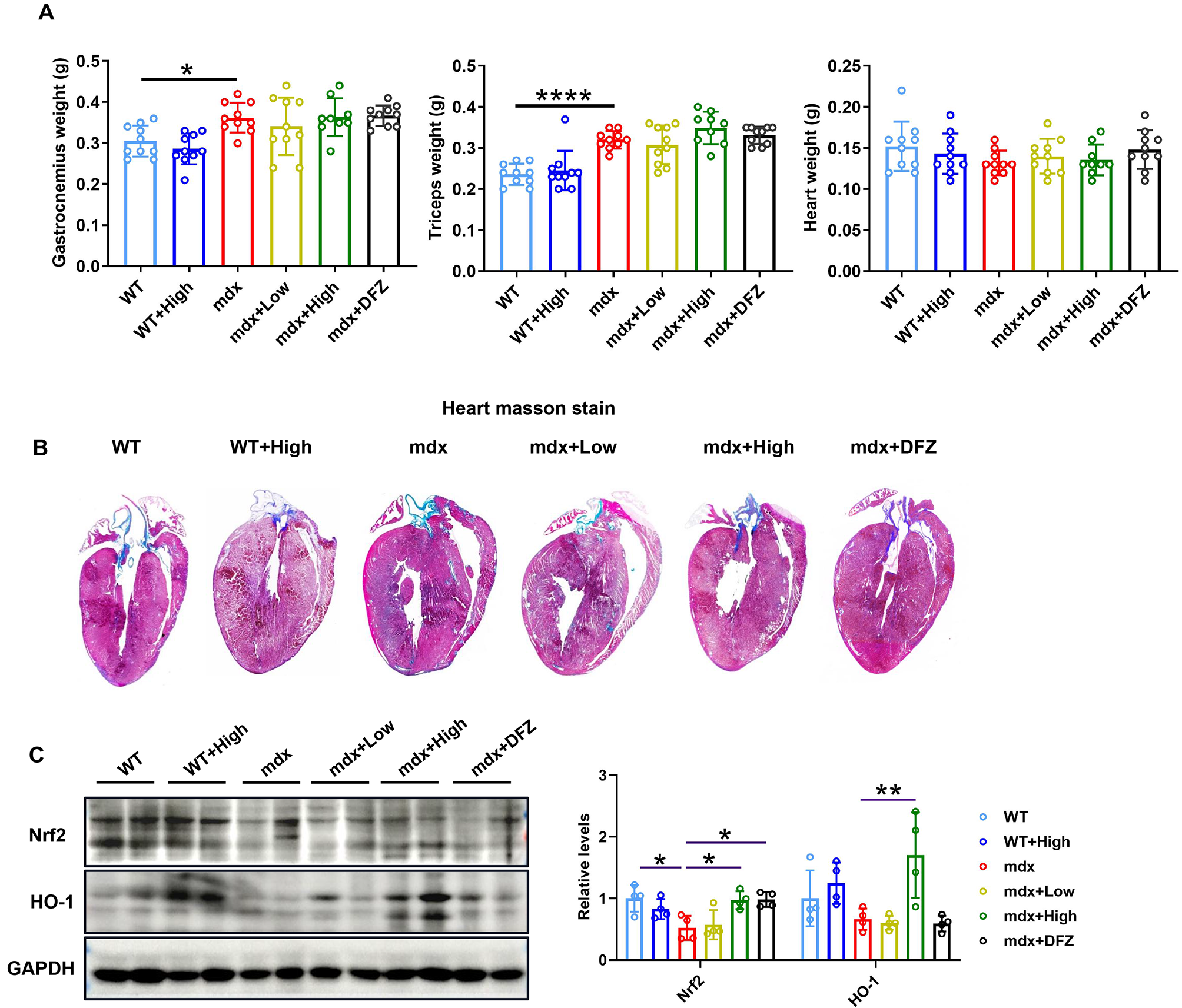

Supplement: Supplementary file 3 — Supplementary Figure 2: The detection of muscle weight, collagen fibres and Nrf2/HO‐1 signal pathway after ARC‐18 treatment in mdx mice. (A) Wet weight statistics of gastrocnemius, triceps and heart. n = 9–10 for each group. (B) Masson Stain for detection of collagen fibres in heart of ARC‐18 treated mdx mice. (C) Western blots analysis of Nrf2 and HO‐1 in gastrocnemius of mdx mice after ARC‐18 administration. n = 4 for each group. Data were shown as mean ± SD. *, p < 0.05, **, p < 0.01. ****, p < 0.0001. [file JCSM-16-e70081-s008.tif]

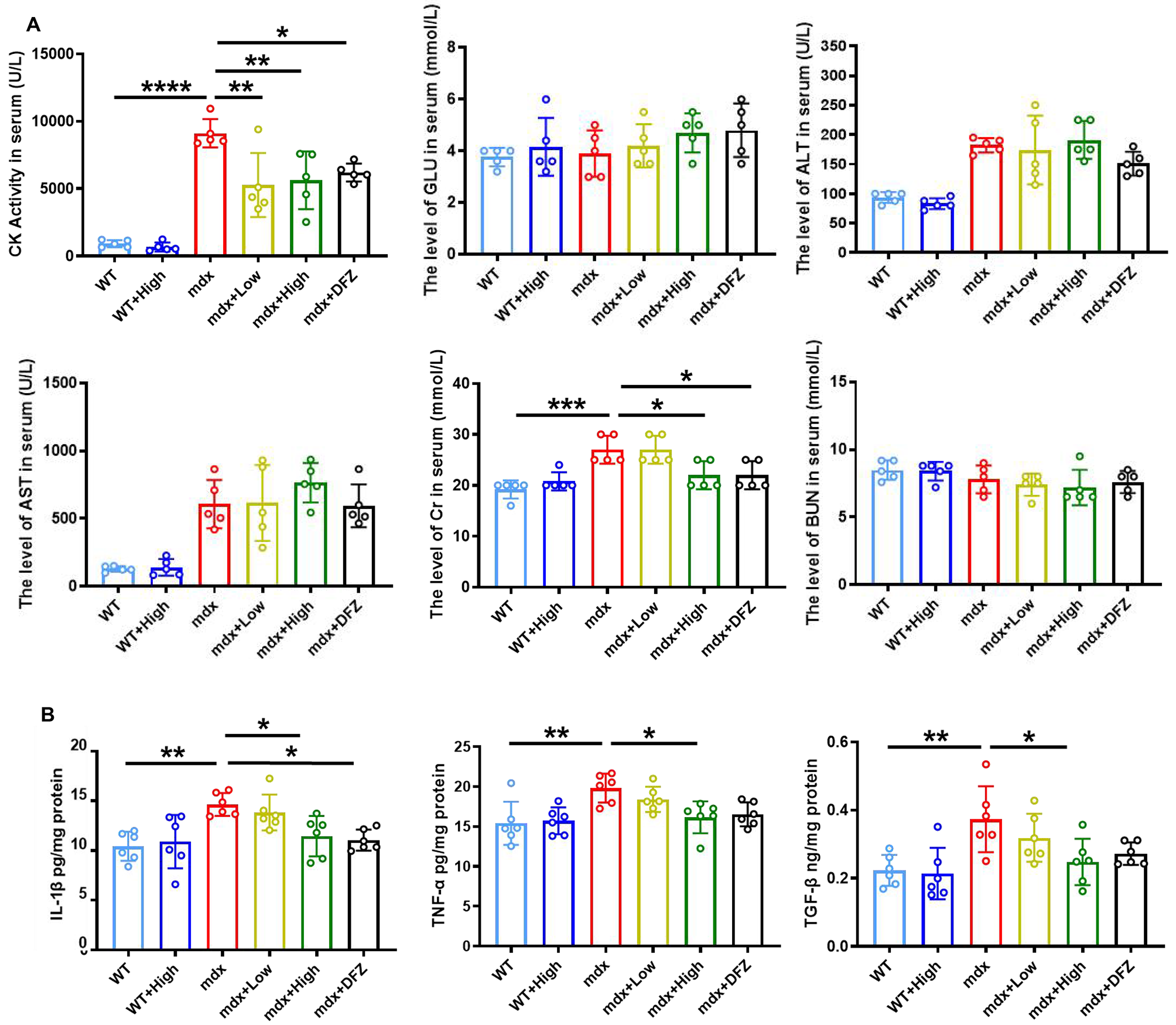

Supplement: Supplementary file 4 — Supplementary Figure 3: ARC‐18 treatment reduced creatine kinase activity and inflammation levels. (A) Blood biochemical analysis of creatine kinase activity, glucose (GLU), alanine aminotransferase (ALT), aspartate aminotransferase (AST), creatinine and blood urea nitrogen (BUN). (B) Elisa analyzed inflammatory of IL‐1β, TNF‐α and TGF β1 in gastrocnemius after ARC‐18 treatment. Data were shown as mean ± SD. *, p < 0.05, **, p < 0.01, ***, p < 0.001, ****, p < 0.0001. n = 5–6 for each group. [file JCSM-16-e70081-s007.tif]

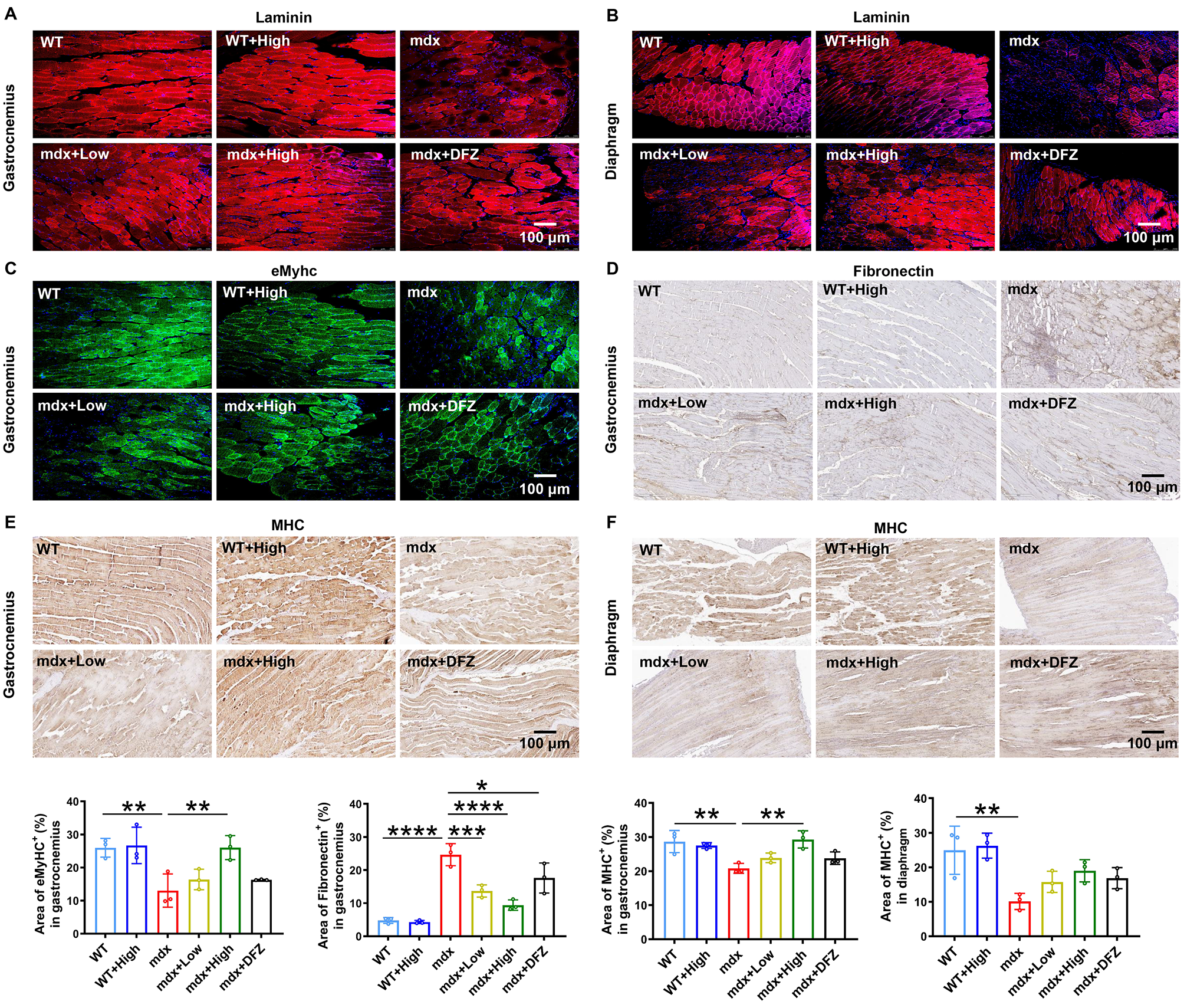

Supplement: Supplementary file 5 — Supplementary Figure 4: ARC‐18 attenuated myoblast differentiation and suppressed fibrosis in mdx mice. Immunofluorescence of the structural protein laminin in gastrocnemius (A) and diaphragm (B). Immunofluorescence and quantification of the regulation of skeletal muscle differentiation protein eMyHC (C). Immunohistochemical and quantification of the collagen fibres composition of Fibronectin in gastrocnemius (D), and the composition of myosin protein MHC in gastrocnemius (E) or in diaphragm (F). Data were shown as mean ± SD. *, p < 0.05, **, p < 0.01, ***, p < 0.001, ****, p < 0.0001. n = 3 for each group. [file JCSM-16-e70081-s003.tif]

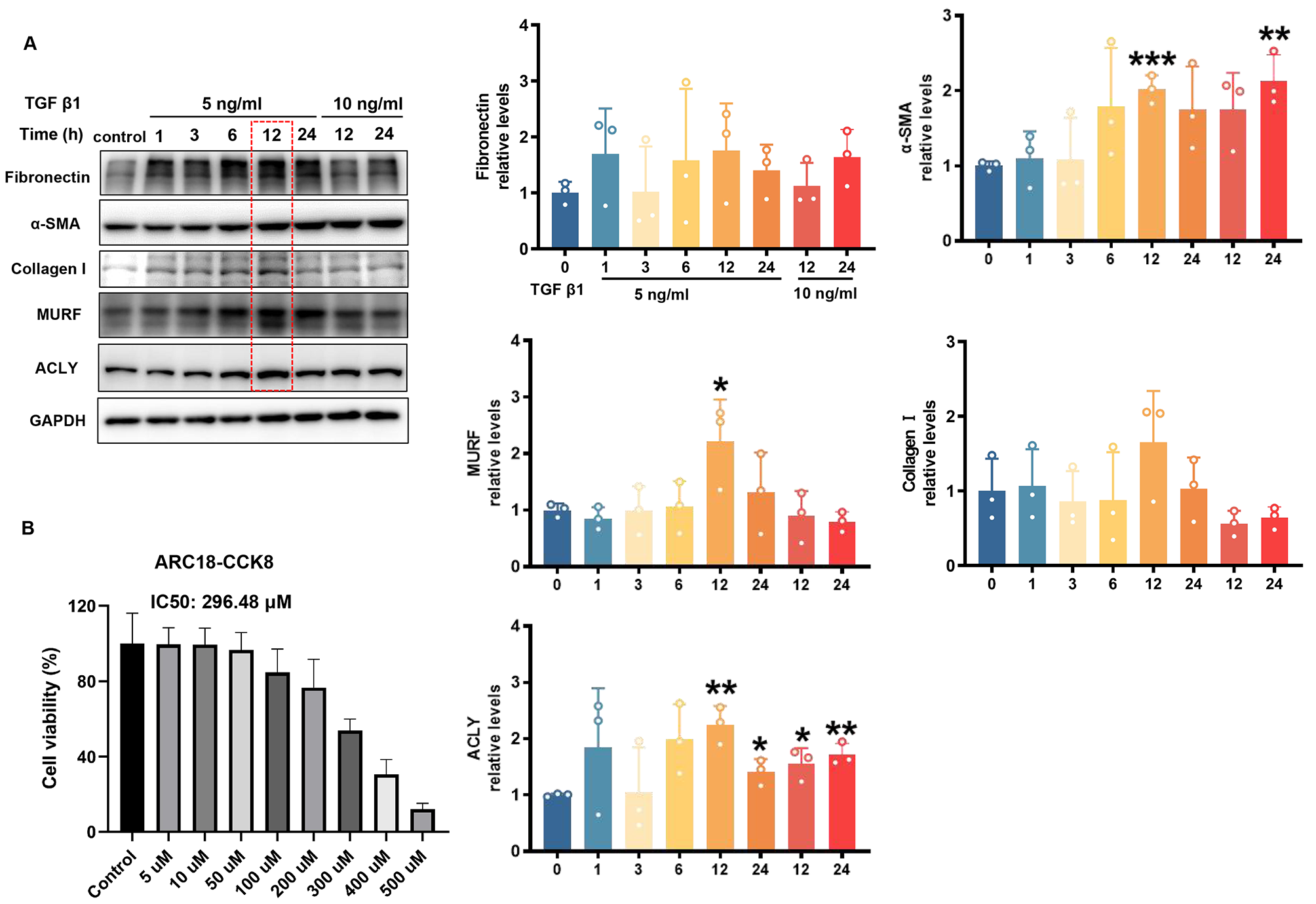

Supplement: Supplementary file 6 — Supplementary Figure 5: TGF‐β1 treatment induced expression of fibrotic protein in C2C12 cell. (A) Western blot and quantification of Fibronectin, Collagen I, MURF and ACLY in C2C12 cell treat with 5 ng/mL or 10 ng/mL TGF‐β1. Data were shown as mean ± SD. *, p < 0.05, **, p < 0.01, ***, p < 0.001, vs. con group. n = 3 for each group. (B) The cell viability of ARC‐18 in C2C12 cell detected by cell counting kit‐8. [file JCSM-16-e70081-s001.tif]

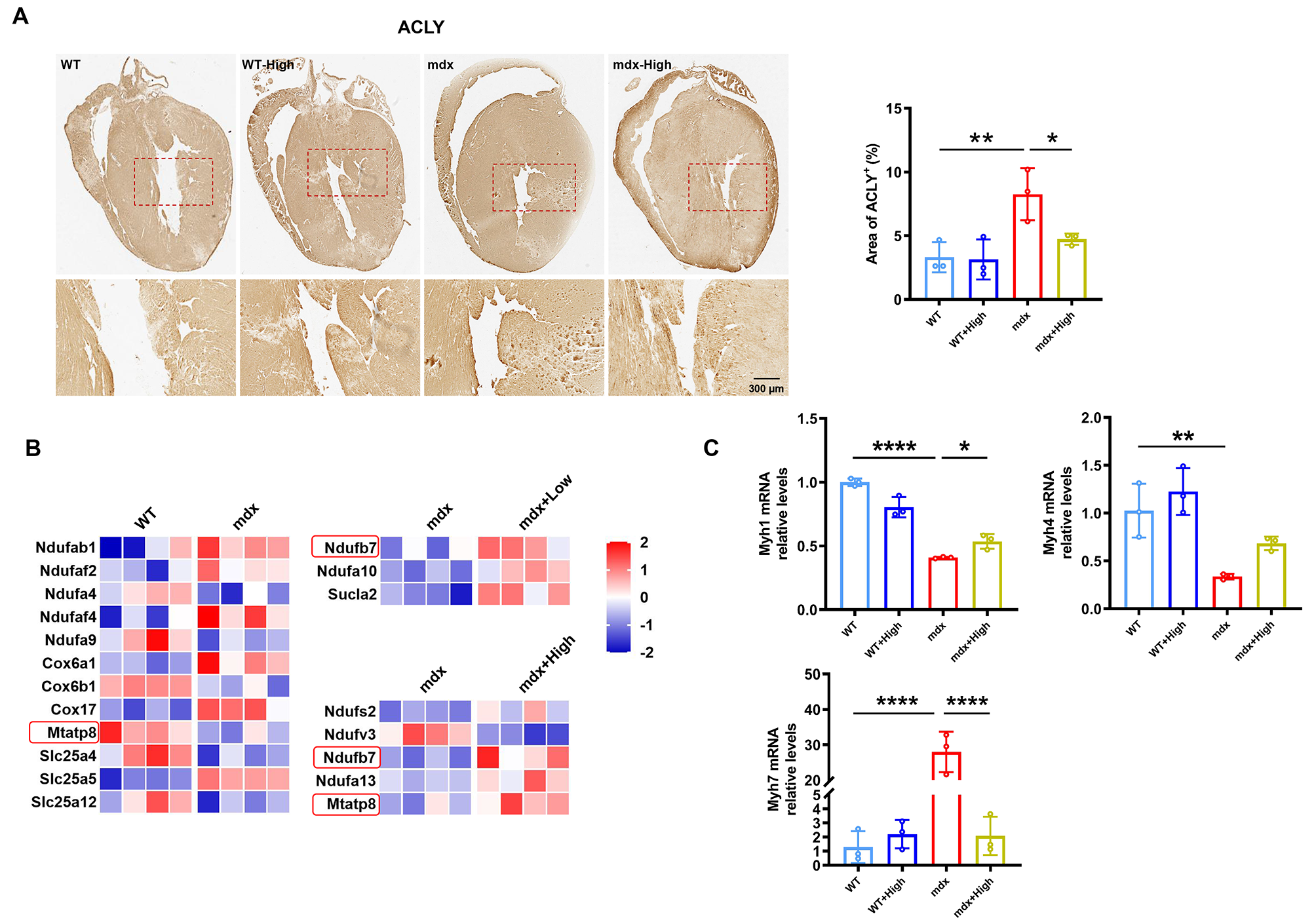

Supplement: Supplementary file 7 — Supplementary Figure 6: ARC‐18 played a protective role in cardiac muscle function, mitochondrial activity and muscle differentiation. (A) Immunohistochemical and quantification of the ACLY in heart of ARC‐18 treated mdx mice. (B) Heatmap of differential protein expression associated with the mitochondrial electron transport chain in different comparison groups. (C) QPCR analysis of Myh1, Myh4 and Myh7 mRNA expression levels in gastrocnemius after ARC‐18 treatment. Data were shown as mean ± SD. *, p < 0.05, **, p < 0.01, ****, p < 0.0001. n = 3 for each group. [file JCSM-16-e70081-s009.tif]

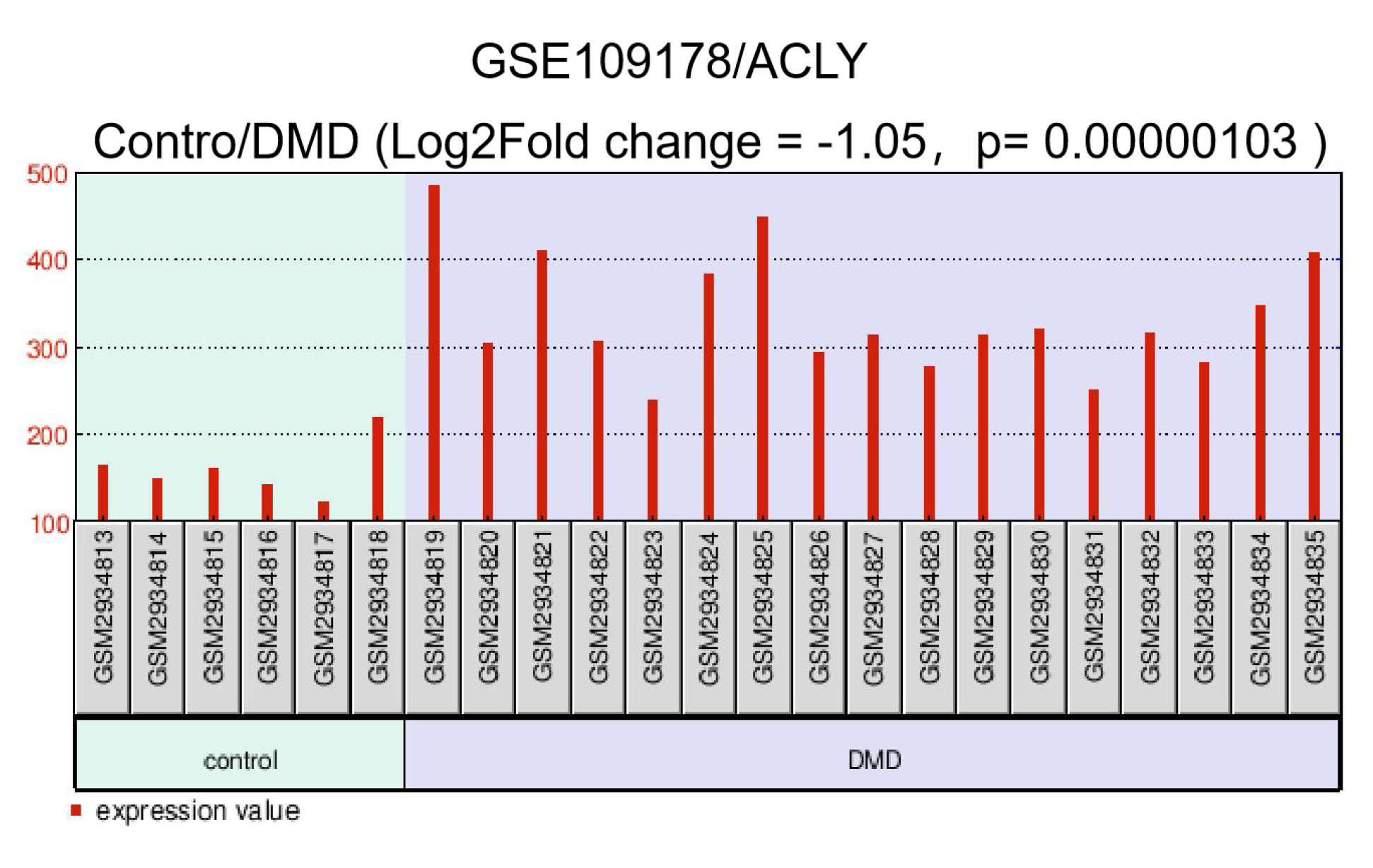

Supplement: Supplementary file 8 — Supplementary Figure 7: ACLY mRNA expression was increased in DMD patient muscles. Bioinformatic analysis of ACLY mRNA expression in publicly available mRNA expression data (GEO Dataset GSE109178), which includes 17 DMD patient and 6 healthy control quadriceps samples. [file JCSM-16-e70081-s002.tif]
